# One-pot synthesis and property study on thieno[3,2-*b*]furan compounds†

**DOI:** 10.1039/c9ra00796b

**Published:** 2019-03-01

**Authors:** Weimin Ma, Jiawei Huang, Chao Li, Yueren Jiang, Baolin Li, Ting Qi, Xiaozhang Zhu

**Affiliations:** School of Chemical Sciences, University of Chinese Academy of Sciences Beijing 100049 P. R. China libl@ucas.ac.cn qiting@ucas.ac.cn; Beijing National Laboratory for Molecular Sciences, CAS Key Laboratory of Organic Solids, Institute of Chemistry, Chinese Academy of Sciences Beijing 100190 P. R. China xzzhu@iccas.ac.cn

## Abstract

Based on the regioselective intermolecular Suzuki coupling and subsequent intramolecular Ullmann C–O coupling reactions, one-pot synthesis of benzo[4,5]thieno[3,2-*b*]benzofurans (BTBFs) was developed after optimization of the reaction conditions including catalysts, solvents, bases, ligands and reaction times. The one-pot reaction, with only 2 mol% Pd(PPh_3_)_4_ and 2 mol% copper(i) thiophene-2-carboxylate (CuTc) as the catalysts, K_3_PO_4_·3H_2_O as the base and *tert*-butanol as the solvent, afforded moderate to good yields (up to 70%) for a variety of substrates.

## Abstract 2

Based on the regioselective intermolecular Suzuki coupling and subsequent intramolecular Ullmann coupling reactions, one-pot synthesis of benzo[4,5]thieno[3,2-*b*]benzofurans (BTBFs) has been developed.

## Graphical abstract

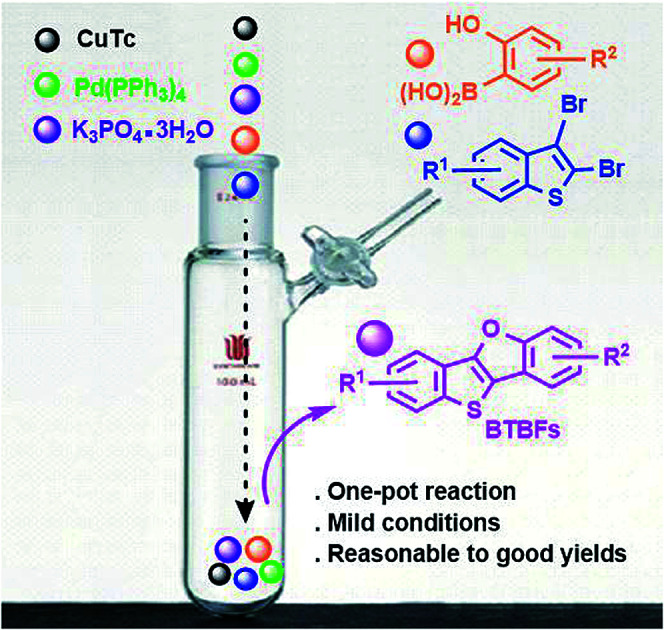

## Introduction

Furan-fused ring compounds exhibit high fluorescence quantum efficiency and high carrier mobility, and thus could be widely used as luminescent materials or hole transporting materials in optoelectronic devices.^1,2^ For instance, benzodifurans (BDFs)^3,4^ and naphthodifurans (NDFs)^5,6^ play an important role in the development of high-performance organic light-emitting diodes (OLEDs) and organic field-effect transistors (OFETs).^2^ Therefore, more and more attention has been paid to the design and synthesis of this type of compounds.^7–15^

Over the years, some synthetic methods including transition metal catalyzed intramolecular C–O coupling^16–18^ and bis-C/H-activated ring closure of diaryl ethers^19,20^ as well as ring closure of *ortho*-hydroxyarylalkynes^21–27^ have been reported for the synthesis of furan-fused ring compounds. These methods are usually high-yielding and applicable to a variety of substrates. Nevertheless, they also suffer from some drawbacks, including the usage of expensive palladium catalysts, cumbersome steps for the preparation of the substrates, and so on. Therefore, these methods are not satisfactory from a synthetic chemistry viewpoint, and new cost-effective synthetic methods for furan-fused ring compounds are urgently needed.

The search for alternative safer, cleaner and environmentally friendly synthetic procedures is one of the major activities in synthetic chemistry. To this end, the reduction of wastes together with the use of renewable feedstocks, environmentally friendly reagents and inexpensive catalysts are important pathways to access more sustainable processes.^28,29^ In this sense, one-pot procedure involving catalytic multistep reactions allows the decrease of energy consuming steps such as separation and purification of intermediates.^30^ These transformations known as tandem, domino or cascade reactions^31,32^ have become an important research area in organic chemistry^33–35^ since they can reduce raw materials and energy consumption and improve atom economy.

**Scheme 1:**
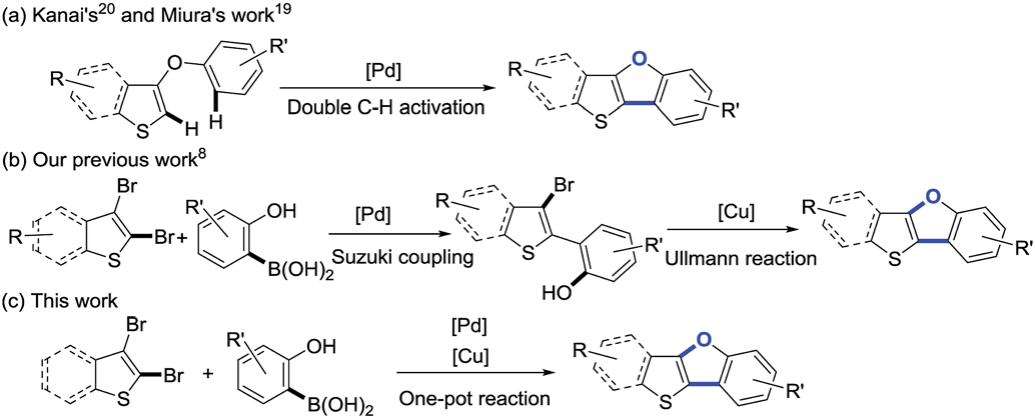
Synthetic strategies toward thieno[3,2-*b*]benzofurans.

As shown in Scheme 1a, thieno[3,2-*b*]benzofurans have been synthesized by palladium-catalyzed double C–H activation from diaryl ethers.^19,20^ However, the reactions were performed under rather hash condition (110 or 150 °C), and the precursor diaryl ethers need to be synthesized by a multiple-step reaction. Recently, our group has reported a new strategy for the synthesis of benzo[4,5]thieno[3,2-*b*]benzofurans (BTBFs) through intramolecular C–O coupling by using cheap copper catalyst (Scheme 1b) under mild condition (90 °C).^8^ We further noticed that both the Suzuki coupling and the subsequent Ullmann reaction require similar reaction conditions: transition metal catalysts, inorganic bases, and the reaction temperature at around 90 °C. In order to further simplify the synthetic route, we thus design one-pot method^30^ to synthesize the target product BTBFs. Fortunately, we successfully achieved this goal after screening the reaction conditions. The yields are moderate to good (up to 70%). Meanwhile, this method shows good functional group compatibility, halogenated compounds are also suitable substrates. On the other hand, we studied the photophysical properties of this type of compounds, representing the first example of thieno[3,2-*b*]furans.

## Results and discussion

Initially, all the reactants and catalysts were added at the same time, during the screening of reaction conditions for the synthesis of BTBF (3a). Unfortunately, we didn't detect the target product. Next, we use the method of adding catalysts in two batches without any other treatment of the intermediate product (*i.e.* Suzuki coupling product). For the first batch, 1a, 2a and the base were mixed in the corresponding solvent and the first catalyst Pd(OAc)_2_ or Pd(PPh_3_)_4_ was added, then the mixture was heated at 90 °C for 24 hours. For the second batch, the second catalyst copper salt was added and heated for another 4 hours. At first, we tried Pd(OAc)_2_ as the catalyst for Suzuki coupling and CuI as the catalyst for Ullmann reaction, and found that no target product was detected (Table 1, entries 1 and 2). Considering that the most commonly used catalyst for Suzuki coupling is Pd(PPh_3_)_4_, we switched to use it as the catalyst in the following condition screening (Table 1, entries 3–24). When the reaction was carried out with 5 mol% CuOAc, CuI, Cu(OAc)_2_ or copper(i) thiophene-2-carboxylate (CuTc) as the catalyst for Ullmann reaction in *N*-methyl pyrrolidone (NMP) (Table 1, entries 5–8) at 90 °C, less than 10% yields of desired product were obtained. Nonetheless, we found that CuTc exhibited the highest catalytic activity. Then the effect of different solvents was evaluated (Table 1, entries 8–10), we found that the three solvents including NMP, 1,4-dioxane and *tert*-butanol afforded essentially the same yields (6.4–6.9%). Considering that *tert*-butanol is more economical and less toxic than the other two solvents, we chose *tert*-butanol as the solvent in the following condition screening. For comparison, we noticed that toxic solvents including toluene, *N*,*N*-dimethylformamide (DMF) or propionic acid were used in previous reports.^8,20^

**Table 1:** Screening of reaction conditions^a^

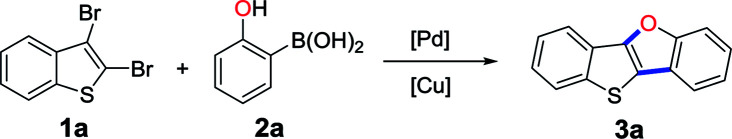						
Entry	Catalyst 1	Catalyst 2	Base	18-crown-6/3 Å M.S.^b^	Solvents	GC yield
1	5 mol% Pd(OAc)_2_	5 mol% CuI	K_2_CO_3_	—/—	1,4-Dioxane	0
2	5 mol% Pd(OAc)_2_	5 mol% CuI	K_2_CO_3_	—/—	DMF	0
3	5 mol% Pd(PPh_3_)_4_	5 mol% CuI	K_2_CO_3_	—/—	1,4-Dioxane	1.1%
4	5 mol% Pd(PPh_3_)_4_	5 mol% CuI	K_2_CO_3_	—/—	DMF	3.7%
5	5 mol% Pd(PPh_3_)_4_	5 mol% CuI	K_2_CO_3_	—/—	NMP	4.6%
6	5 mol% Pd(PPh_3_)_4_	5 mol% CuOAc	K_2_CO_3_	—/—	NMP	5.6%
7	5 mol% Pd(PPh_3_)_4_	5 mol% Cu(OAc)_2_	K_2_CO_3_	—/—	NMP	5.1%
8	5 mol% Pd(PPh_3_)_4_	5 mol% CuTc	K_2_CO_3_	—/—	NMP	6.8%
9	5 mol% Pd(PPh_3_)_4_	5 mol% CuTc	K_2_CO_3_	—/—	1,4-Dioxane	6.9%
10	5 mol% Pd(PPh_3_)_4_	5 mol% CuTc	K_2_CO_3_	—/—	*t*-BuOH	6.4%
11	5 mol% Pd(PPh_3_)_4_	5 mol% CuTc	Cs_2_CO_3_	—/—	*t*-BuOH	10%
12	5 mol% Pd(PPh_3_)_4_	5 mol% CuTc	K_3_PO_4_	—/—	*t*-BuOH	29%
13	5 mol% Pd(PPh_3_)_4_	5 mol% CuTc	K_3_PO_4_	—/—	*t*-BuOH/10 μL H_2_O^c^	42%
14	5 mol% Pd(PPh_3_)_4_	5 mol% CuTc	K_3_PO_4_	—/—	*t*-BuOH/0.1 mL H_2_O^c^	35%
15	5 mol% Pd(PPh_3_)_4_	5 mol% CuTc	K_3_PO_4_	—/—	*t*-BuOH/1 mL H_2_O^c^	25%
16	5 mol% Pd(PPh_3_)_4_	5 mol% CuTc	K_3_PO_4_·3H_2_O	—/—	*t*-BuOH	33%
17	5 mol% Pd(PPh_3_)_4_	5 mol% CuTc	K_3_PO_4_·3H_2_O	5 mol%/—	*t*-BuOH	36%
18	5 mol% Pd(PPh_3_)_4_	5 mol% CuTc	K_3_PO_4_·3H_2_O	—/200 mg	*t*-BuOH	43%
19	5 mol% Pd(PPh_3_)_4_	5 mol% CuTc	K_3_PO_4_·3H_2_O	5 mol%/200 mg	*t*-BuOH	49%
20	2 mol% Pd(PPh_3_)_4_	5 mol% CuTc	K_3_PO_4_·3H_2_O	5 mol%/200 mg	*t*-BuOH	56%
21	0.5 mol% Pd(PPh_3_)_4_	5 mol% CuTc	K_3_PO_4_·3H_2_O	5 mol%/200 mg	*t*-BuOH	46%
22	5 mol% Pd(PPh_3_)_4_	2 mol% CuTc	K_3_PO_4_·3H_2_O	5 mol%/200 mg	*t*-BuOH	43%
23	2 mol% Pd(PPh_3_)_4_	2 mol% CuTc	K_3_PO_4_·3H_2_O	5 mol%/200 mg	*t*-BuOH	68% (62%)^d^
**24**	**2 mol% Pd(PPh_3_)_4_**	**2 mol% CuTc**	**K_3_PO_4_·3H_2_O**	**5 mol%/200 mg**	***t*-BuOH**	**77% (70%)^d^^,^^e^**

a Reaction conditions: the mixture of 1a (140 mg, 0.48 mmol), 1b (55 mg, 0.4 mmol), the first catalyst Pd(PPh_3_)_4_ and the base in the solvent (5.0 mL) under N_2_ was stirred at 90 °C for 24 hours. Then the second copper catalyst was added and heated for another 4 hours. 1,1,2,2-Tetrachloroethane was used as an internal standard to determine the GC yield.^36^

b 3 Å molecular sieves were activated under vacuum condition at 250 °C for 5 hours before being used as water absorbent.

c Different amount of water was added as additive.

d The yield in parentheses is the isolated yield.

e 2-Hydroxyphenylboronic acid (2a) was replaced by 2-hydroxyphenylboronic acid pinacol ester.

We next screened a series of bases (Table 1, entries 10–12) and found that K_3_PO_4_ showed the best results (29% yield, Table 1, entry 12). After the screening of solvents and bases, we also explored the effect of the amount of water on reaction yield (Table 1, entries 13–15). To our delight, significant increase of the yield was observed when the reaction was carried out in 5 mL *tert*-butanol with 10 μL water (Table 1, entry 13). However, addition of excess amount of water reduces the reaction yield (Table 1, entry 15). This could be explained as followed: addition of small amount of water could increase the reaction rate of Suzuki coupling reaction since water and base are required to activate the boronic acid,^37,38^ and it doesn't do harm to the Ullmann reaction; while addition of large amount of water significantly reduces the catalytic activity of copper catalyst, thus does harm to the Ullmann reaction heavily and the intermediate remained when the reaction mixture was worked up. In order to simplify the experimental operation, we used K_3_PO_4_·3H_2_O as the source of base and water to avoid the need of addition of base and water separately (Table 1, entries 16–24).

On the other hand, we found that addition of 18-crown-6 as an additive to the reaction system can increase the solubility of K_3_PO_4_·3H_2_O^37,39^ in *tert*-butanol thus increase the reaction rate and improve the reaction yield (36%, Table 1, entry 17). It is well known that coordinated water in the base favors the previous Suzuki coupling,^37^ however, water will also coordinate with the copper catalyst in the subsequent Ullmann reaction and reduce its catalytic activity. Therefore, we introduced activated 3 Å molecular sieves as water absorbent into the reaction system after the Suzuki coupling has been carried out, and thus reaction yield has been further improved to 43% (Table 1, entry 18). By further screening the amount of two catalysts (Table 1, entries 19–23), we obtained the highest GC yield of 68% (isolated yield 62%, Table 1, entry 23). It is noted that the amount of two catalysts could be reduced to 2 mol%, which is significantly lower than our previous report [5 mol% Pd(PPh_3_)_4_, 10 mol% CuI]^8^ and the others' reports.^19,20^ Strikingly, when we used 2-hydroxyphenyl-boronic acid pinacol ester instead of 2-hydroxybenzeneboronic acid (Table 1, entry 24), GC yield can be further increased to 77% (isolated yield 70%), which is an improvement over the previously reported yield (67% total yield *via* two steps).

With the optimal reaction condition in hand, we next explored reaction scope. The reaction proved to be robust and versatile and allowed the synthesis of a variety of BTBFs as shown in Table 2. It worked well for various substrates bearing either electron-withdrawing or electron-donating groups on the aromatic rings, such as F, Cl, Me, C_8_H_17_, or *t*-Bu, affording the desired products in moderate to good yields (Table 2, 3a–3i). The yields are not high, it is understandable considering that both the Suzuki coupling and the subsequent Ullmann reaction are usually not very high yielding. To our delight, for the known compounds (3a–3b), we achieved higher yield than previous reports (70% *vs.* 67%, 61% for 3a; 60% *vs.* 46% for 3b).^8,20^ Furthermore, the π-extended product BTNF (3j) could be obtained through this approach in moderate yield (35%). This can extend the conjugation of this type of compounds and change their photophysical properties.

**Table 2:** Substrates scope^a^

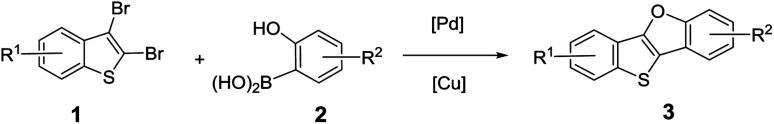
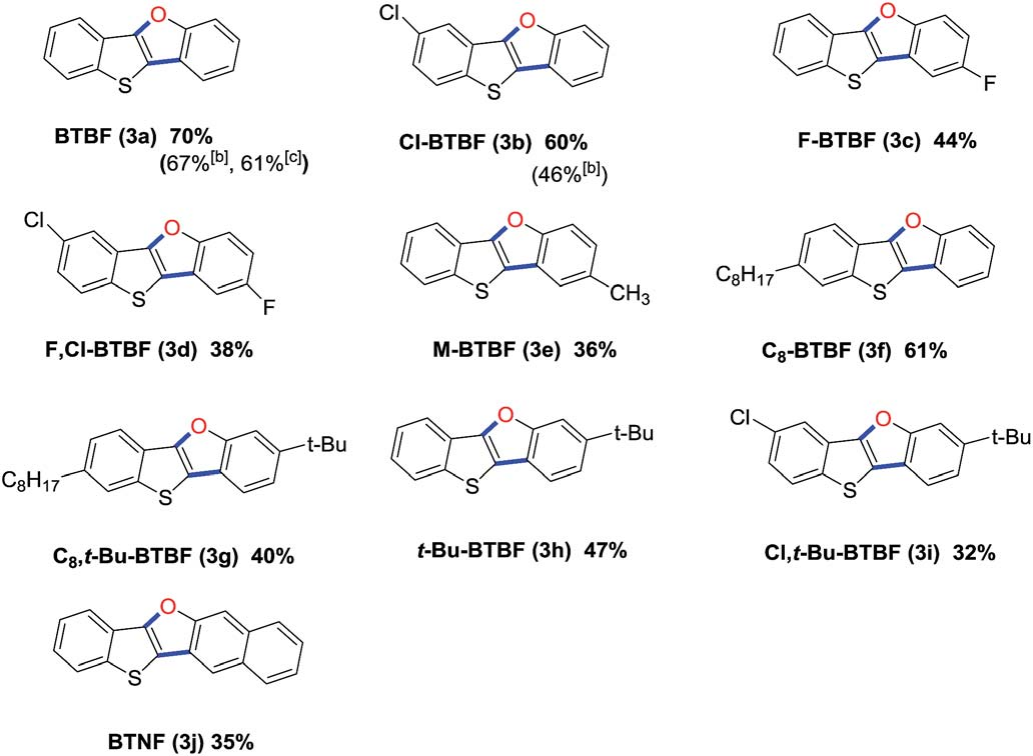

a Reactions were conducted on 1 (0.48 mmol), 2 (0.4 mmol) and K_3_PO_4_·3H_2_O (319 mg, 1.2 mmol), and all the yields are isolated ones.

b Total yields *via* two steps in our previous report.^8^

c Isolated yield reported by Kanai group.^20^

**Fig. 1:**
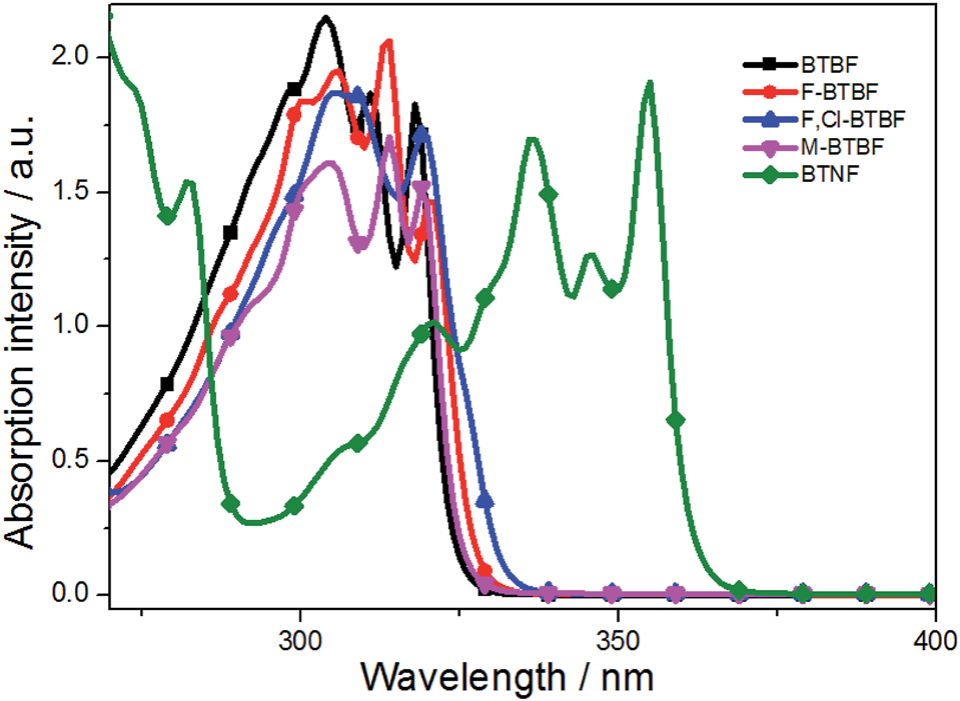
UV-Vis absorption spectra in ethanol (6 × 10^−5^ mol L^−1^).

The UV-Vis absorption and fluorescence spectra of BTBF (3a), F-BTBF (3c), F,Cl-BTBF (3d), M-BTBF (3e), and BTNF (3j) in dilute ethanol solutions were investigated. As shown in Fig. 1, the absorption spectra of these compounds exhibits fine structures, and the maximum absorption peaks at 318, 320, 319, 319 and 355 nm respectively could be assigned to π–π* transitions of the conjugated backbones containing sulfur and oxygen heteroatoms.^40,41^ We can see that either halogen atoms on the F-BTBF and F,Cl-BTBF molecules or methyl group in the M-BTBF molecule have neglectable effect on the maximum absorption. But for BTNF, the introduction of naphthalene ring extends the conjugated system when compared to BTBFs, leading to a significant red shift of absorption peak.

**Fig. 2:**
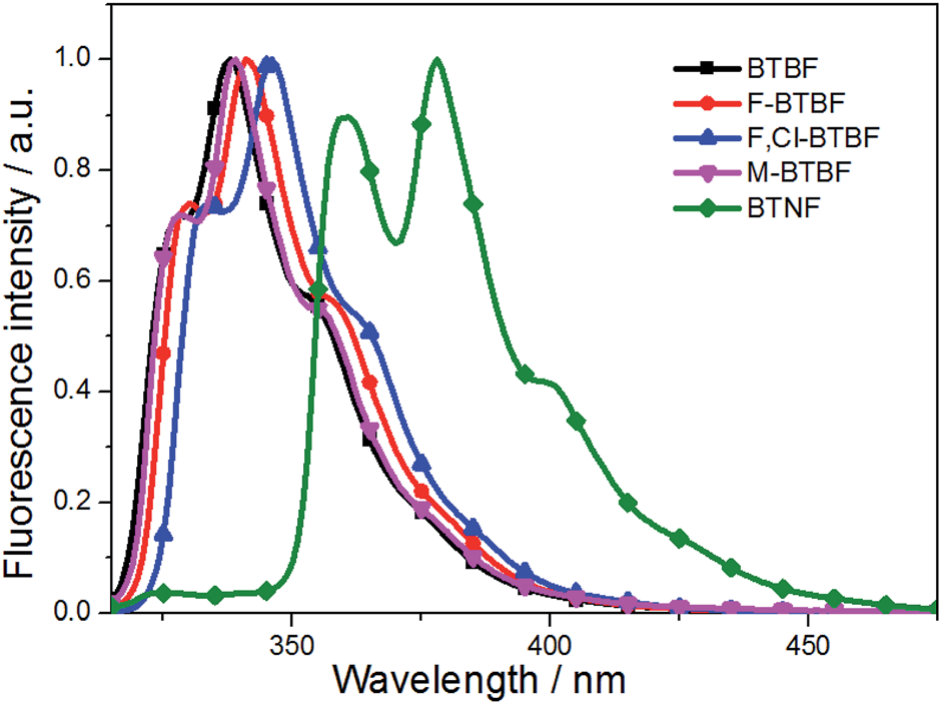
Fluorescence spectra in ethanol (2 × 10^−7^ mol L^−1^).

As shown in Fig. 2, the maximum emission peaks of these compounds are located at 339, 342, 347, 340, 378 nm, respectively. The fluorescence quantum yields (*Φ*_f_) of the compounds were determined to be 0.01, 0.03, 0.04, 0.10, 0.72 respectively in dilute ethanol solutions, with 9,10-diphenylanthracene (*Φ*_f_ = 0.95)^42^ as the reference standard. To be noted, BTNF exhibits significantly red-shifted fluorescence peak at 378 nm and much increased fluorescence quantum yield due to its extended π system provided by the rigid naphthalene ring. The introduction of rigid naphthalene ring may reduce the nonradiative decay rate, resulting in high fluorescence quantum yield. Thus, it is expected to be a good luminescent material. To our best knowledge, this is the first example of study on photophysical properties of thieno[3,2-*b*]furans.

To understand the frontier molecular orbitals and spectroscopic properties of these five compounds, the computation of molecular orbital geometries were performed by density functional theory (DFT) at the B3LYP/6-31G level of theory.^43,44^ In Fig. S2,† we can see that the highest occupied molecular orbitals (HOMOs) and lowest occupied molecular orbitals (LUMOs) of these compounds are homogeneously delocalized over the entire backbone. The HOMO levels (*E*_HOMOs_) of BTBF, F-BTBF, F,Cl-BTBF, M-BTBF and BTNF are calculated to be −5.71, −5.94, −6.22, −5.66, −5.45 eV, respectively, and the LUMO levels (*E*_LUMOs_) are −1.29, −1.54, −1.82, −1.24, −1.64 eV, respectively (Fig. S3†).

Compared with BTBF, F-BTBF and F,Cl-BTBF show much deeper HOMO and LUMO energy levels resulting from the existence of electron-withdrawing halogen atoms (Fig. S3†). On the contrary, the introduction of an electron-donating methyl group slightly raises the HOMO and LUMO energy levels of M-BTBF. Obviously, the tendency of the calculated energy gaps of these compounds are in agreement with those of the energy gaps calculated from the UV absorption onset (Table 3 and Fig. S3†).

**Table 3:** Photophysical properties of BTBFs and BTNF

Compounds	*λ*_abs,max_ (nm)	*E*_g_ a (ev)	*λ*_FL_ (nm)	Stoke shift (nm)	*Φ*_f_ b
BTBF (3a)	318	3.79	339	21	0.01
F-BTBF (3c)	320	3.73	342	22	0.03
F,Cl-BTBF (3d)	319	3.75	347	28	0.04
M-BTBF (3e)	319	3.77	340	21	0.10
BTNF (3j)	355	3.41	378	23	0.72

a The optical band gap E_g_ is calculated by the formula E_g_ = 1240/*λ*_onset_. *λ*_onset_ represents the largest edge of the UV-Visible absorption spectrum.

b 9,10-Diphenylanthracene (*Φ*_f_ = 0.95) as a reference in ethanol.^42^

## Conclusions

In summary, we have developed a one-pot method for the construction of ladder-type BTBFs. One-pot method can not only eliminate the requirement of separation of the intermediate product, but also reduce the use of various solvents, in line with the concept of green chemistry. In addition, low loading of palladium and copper catalysts and mild reaction temperature make this strategy more economic and attractive. On the other hand, photophysical property study showed that π-extended BTNF exhibits high quantum efficiency. We believe the simple synthesis of BTBFs in one-pot method makes it more convenient to prepare these compounds and will promote their application as semiconductor materials.

## Conflicts of interest

There are no conflicts to declare.

## Supplementary Material

RA-009-C9RA00796B-s001
