# Supplementary material for: One-pot synthesis and property study on thieno[3,2-b]furan compounds
Source: RSC Adv. 2019 Mar 1;9(13):7123–7. doi: 10.1039/c9ra00796b (PMC9061063; doi:10.1039/c9ra00796b)
Supplement: RA-009-C9RA00796B-s001 [file RA-009-C9RA00796B-s001.pdf]

Electronic Supplementary Material (ESI) for RSC Advances.
This journal is © The Royal Society of Chemistry 2019

Supporting Information

One-pot Synthesis and Property Study on Thieno[3,2-*b*]furan Compounds

Weimin Ma,[a] Jiawei Huang,[a] Chao Li,[a] Yueren Jiang,[a] Baolin Li,[a,*] Ting Qi,[a,*] Xiaozhang Zhu[b,*]

[a] School of Chemical Sciences, University of Chinese Academy of Sciences, Beijing 100049, P. R. China

[b] Beijing National Laboratory for Molecular Sciences, CAS Key Laboratory of Organic Solids, Institute of Chemistry, Chinese Academy of Sciences, Beijing 100190, P. R. China

Table of Contents

1. GC standard curve of BTBF…………………………………………………. ...S2

2. DFT calculations……………………………………………………………...S3

3. Experimental section…………………………………………………............S9

4. Measurement of photophysical data……………………………………………S15

5. ^{1}H and ^{13}C NMR spectra and HRMS spectra……………………. ….………....S16

6. References…………………………………………………………………….S38

1. GC Standard curve of BTBF

Table S1. GC standard curve of BTBF with 1,1,2,2-tetrachloroethane (TCE).

GC Method: 50 °C hold for 2 min, followed by a temperature increase of 20 °C / min to 300 °C, hold 3 min (total run time: 17.5 min). Retention time: TCE 4.69 min; BTBF 12.86 min.

Entry	Mass of BTBF (mg)	Mass of TCE (mg)	$y = \frac{Mass\ of\ BTBF}{Mass\ of\ TCE}$	GC Area of BTBF	GC Area of TCE	$x = \frac{Area\ of\ BTBF}{Area\ of\ TCE}$
1	0	0	0	0	0	0
2	0.075	160	0.00046875	20717	12180674	0.001700809
3	0.15	160	0.0009375	36760	9931586	0.003701322
4	0.225	160	0.00140625	38648	7151579	0.005404121
5	0.3	160	0.001875	53975	7461543	0.007233758
6	0.375	160	0.00234375	74013	8133414	0.009099869

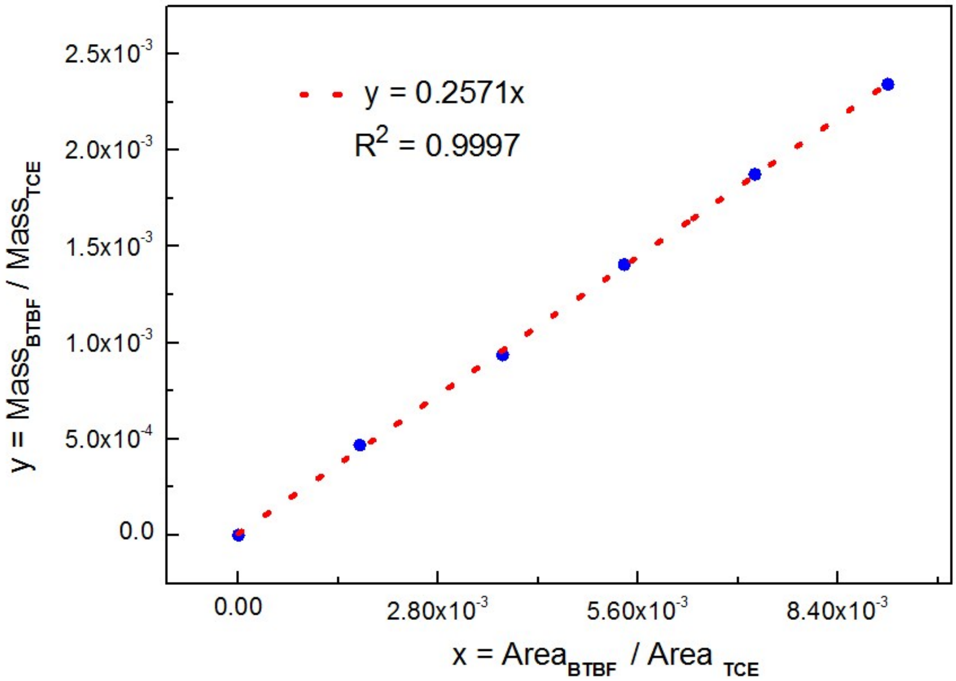

Figure S1. The standard curve of BTBF with TCE as an internal reference.[1]

2. DFT calculations [2]

DFT calculation of BTBF **(3a)**

Table S2. B3LYP/6-31G optimized standard orientation of BTBF **(3a)**.

Atom	X	Y	Z
C	4.36895612	0.14128040	0.00021046
C	3.86527687	1.45544452	0.00003437
C	2.49206350	1.69436855	-0.00009193
C	1.60556944	0.60272821	-0.00002395
C	2.13049073	-0.71911233	0.00013641
C	3.50283266	-0.95765756	0.00025835
C	0.17743775	0.55610888	-0.00014655
C	-0.41776321	-0.68024402	-0.00006972
S	0.81012717	-2.01162283	0.00014675
O	-0.77354594	1.58211870	-0.00052399
C	-2.01955807	0.92143699	-0.00012229
C	-1.84328106	-0.48987468	-0.00013359
C	-3.26119658	1.53883765	0.00001216
C	-4.38096038	0.69862965	0.00010660
C	-4.24094028	-0.70417851	0.00006707
C	-2.98259724	-1.31015710	-0.00004763
H	5.44103080	-0.02509812	0.00030129
H	4.55511398	2.29273599	-0.00000896
H	2.10090338	2.70568888	-0.00023432
H	3.89275555	-1.96957149	0.00038224
H	-3.34973380	2.61825883	-0.00004977
H	-5.37327833	1.13650570	0.00016748
H	-5.13084464	-1.32475937	0.00015145
H	-2.88759559	-2.39040856	-0.00000407

DFT calculation of F-BTBF **(3c)**

Table S3. B3LYP/6-31G optimized standard orientation of F-BTBF **(3c)**.

Atom	X	Y	Z
C	1.99322160	0.56881678	-0.00006849
C	2.42910857	-0.78470963	0.00004425
C	3.78274872	-1.11316185	0.00010013
C	4.71890429	-0.07334581	0.00002496
C	4.30343902	1.27137913	-0.00007423
C	2.94912648	1.60020930	-0.00010162
C	0.56528890	0.61629756	-0.00004920
C	-0.10934973	-0.57949759	-0.00001164
H	4.10574160	-2.14828199	0.00022768
H	5.77761166	-0.31011324	0.00006284
H	5.04702960	2.06118083	-0.00012845
H	2.62540621	2.63507940	-0.00014736
C	-3.89612599	-0.33300540	-0.00016067
C	-2.70424527	-1.04424080	-0.00026168
C	-1.51741851	-0.29488476	-0.00015969
C	-1.60092206	1.12597358	-0.00008532
C	-2.80000857	1.82337208	0.00018058
C	-3.97583993	1.06573331	0.00011014
H	-2.70999887	-2.12635423	-0.00038132
H	-2.81951464	2.90572368	0.00039655
H	-4.94889109	1.53967452	0.00043954
O	-0.31472870	1.70230213	0.00005429
S	1.02701852	-1.98851820	0.00009850
F	-5.09104625	-1.05029448	0.00006612

DFT calculation of F,Cl-BTBF **(3d)**

Table S4. B3LYP/6-31G optimized standard orientation of F,Cl-BTBF **(3d)**.

Atom	X	Y	Z
C	-1.38145731	0.05133421	-0.00002335
C	-1.59779874	-1.35411568	0.00006672
C	-2.88388238	-1.88914131	0.00044388
C	-3.97663227	-1.01573103	0.00037199
C	-3.75274842	0.36734640	0.00011163
C	-2.48310981	0.92557362	-0.00023198
C	0.01961649	0.32636114	-0.00003111
C	0.87360141	-0.74877902	-0.00020283
H	-3.04505080	-2.96107076	0.00051414
H	-4.98805022	-1.40188925	0.00042294
H	-2.33910024	1.99837396	-0.00045829
C	4.43089944	1.48562114	0.00001967
C	4.57348345	0.09168148	-0.00003151
C	3.50898481	-0.79879618	-0.00007036
C	2.21922453	-0.24558086	-0.00019265
C	2.07684262	1.17055185	-0.00012286
C	3.15061264	2.04874477	-0.00001571
H	5.31668171	2.10749251	0.00008457
H	3.68633371	-1.86629914	-0.00013237
H	2.99916032	3.12058002	0.00000575
O	0.71536398	1.53674469	0.00019009
S	-0.02539247	-2.31949350	-0.00030785
F	5.86572189	-0.42778418	0.00037486
Cl	-5.20023075	1.48119914	-0.00005616

DFT calculation of M-BTBF **(3e)**

Table S5. B3LYP/6-31G optimized standard orientation of M-BTBF **(3e)**.

Atom	X	Y	Z
C	2.02184757	0.55916418	-0.00005499
C	2.44163328	-0.79969524	0.00001991
C	3.79101255	-1.14488516	0.00011544
C	4.74082116	-0.11726992	0.00016289
C	4.34175340	1.23227607	0.00009922
C	2.99138945	1.57784902	-0.00001288
C	0.59453779	0.62499042	-0.00016256
C	-0.09601541	-0.56120458	-0.00018100
H	4.10064275	-2.18419398	0.00016454
H	5.79652747	-0.36741263	0.00025312
H	5.09498988	2.01305992	0.00013210
H	2.68073711	2.61676956	-0.00007044
C	-3.93739670	1.10803129	0.00009828
C	-3.92398532	-0.30903059	0.00007990
C	-2.70081874	-0.99049442	-0.00001463
C	-1.50212657	-0.25955919	-0.00008017
C	-1.56637256	1.15851206	-0.00004460
C	-2.76112536	1.86266797	0.00003487
H	-4.89315531	1.62334729	0.00021028
H	-2.68266636	-2.07547909	-0.00010293
H	-2.77380505	2.94575503	0.00005093
O	-0.27273033	1.72200353	-0.00012050
C	-5.23221611	-1.07153231	0.00012946
H	-5.06258749	-2.15257202	0.00065843
H	-5.83754442	-0.82877356	0.88262296
H	-5.83710335	-0.82964495	-0.88290478
S	1.02363605	-1.98473758	-0.00007406

DFT calculation of BTNF **(3j)**

Table S6. B3LYP/6-31G optimized standard orientation of BTNF **(3j)**.

Atom	X	Y	Z
C	-2.76976957	0.59410781	-0.00002392
C	-3.27470436	-0.73556589	0.00012537
C	-4.64341135	-0.99439766	0.00035385
C	-5.52522851	0.09189557	0.00045317
C	-5.04126658	1.41377824	0.00032510
C	-3.67207958	1.67311378	0.00008318
C	-1.34175178	0.57027159	-0.00030296
C	-0.72908677	-0.65566479	-0.00037655
H	-5.01865602	-2.01180849	0.00046048
H	-6.59474399	-0.09018030	0.00063351
H	-5.74370605	2.24049811	0.00040556
H	-3.29540867	2.68989385	-0.00002320
C	5.54882627	-0.82952447	0.00039471
C	4.30499347	-1.42316174	0.00010562
C	3.11181249	-0.63937574	-0.00010221
C	3.23423757	0.80286575	0.00001190
C	4.53781362	1.37910020	0.00031742
C	5.66657487	0.58682093	0.00050258
H	1.75002898	-2.32925800	-0.00023993
H	6.44496354	-1.44158742	0.00053807
H	4.21269781	-2.50555952	0.00002105
C	1.82775586	-1.24691826	-0.00034140
C	2.06385009	1.61441400	-0.00017040
H	4.62631442	2.46172213	0.00039960
H	6.65147745	1.04252442	0.00073564
C	0.85127409	0.98129327	-0.00051619
C	0.69566639	-0.44920722	-0.00044881
H	2.13704082	2.69554716	-0.00012832
O	-0.41136396	1.61820638	-0.00047977
S	-1.93450837	-2.00628220	-0.00008169

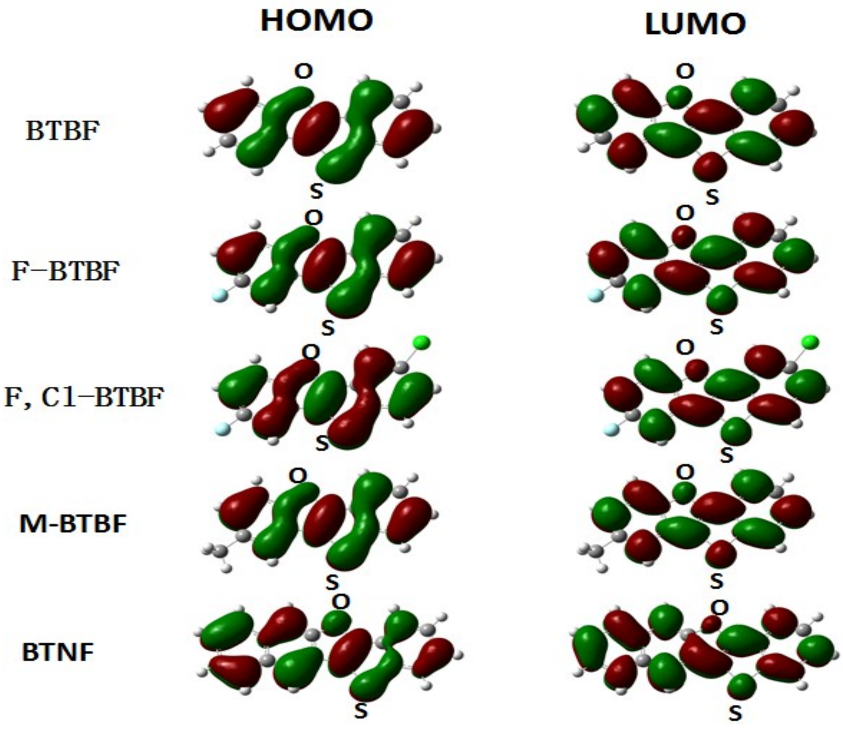

Figure S2. HOMOs and LUMOs of BTBF (**3a**), F-BTBF (**3c**), F,Cl-BTBF (**3d**), M-BTBF (**3e**) and BTNF (**3j**) by DFT calculations at the B3LYP/6-31G level.

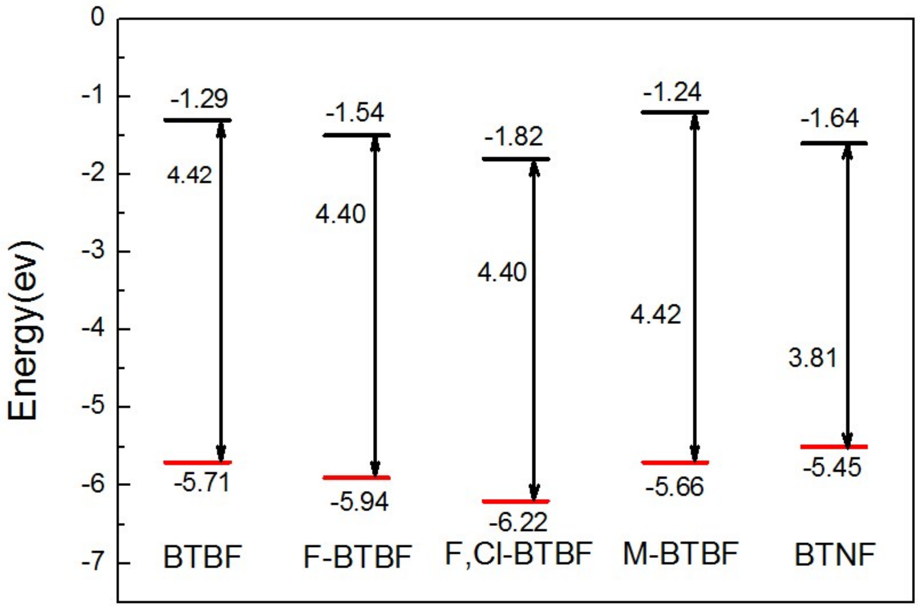

Figure S3. Energy level (E_{HOMOs} and E_{LUMOs}) diagrams of BTBFs and BTNF.

3. Experimental section

General information

All reagents were purchased from commercial suppliers and used without further purification unless otherwise specified. Thin layer chromatography (TLC) was performed using TLC silica gel 60 F254 glass plates. Silica gel 60 (200-300 mesh) was used for column chromatography. Proton nuclear magnetic resonance (^{1}H NMR) and carbon nuclear magnetic resonance (^{13}C NMR) spectra were measured on JEOL 400YH spectrometer. Chemical shifts for hydrogens are reported in parts per million (ppm, δ scale) downfield from tetramethylsilane (δ = 0), multiplicity (s = singlet, d = doublet, t = triplet, q = quartet, m = multiplate), and coupling constant (Hz). Detection and analysis of compounds by gas chromatography (Shimadzu GC-2010 plus). High resolution mass spectra (HRMS) were determined on a Bruker Apex IV Fourier Transform Mass Spectrometer (EI). UV-Vis spectra were measured by Shimadzu UV-1800 ENG240V, SOFT. Fluorescence spectra were measured by Shimadzu-RF-5301pc.

Synthesis and characterization of related compounds:

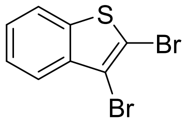

2,3-Dibromobenzo[*b*]thiophene:

Benzo[*b*]thiophene (5.4 g, 40 mmol) was dissolved in 50 ml $CHCl_3$, and then Br_2 (4.3 ml, 84 mmol) was added to the solution under ice-water bath. After it was stirred for 18 h at room temperature, $NaHCO_3$ aqueous solution was added to neutralize hydrobromic acid formed in the reaction mixture. The organic layer was extracted with dichloromethane (DCM) and washed with saturated brine, and then dried with $MgSO_4$. The solvents were removed and the residue was recrystallized from methanol/ethanol co-solvents (v/v = 2/1), affording 2,3-dibromobenzo[*b*]thiophene (10 g, 34 mmol, 85%) as white solid. ^{1}H NMR (400 MHz, $CDCl_3$): δ 7.76-7.71 (m, 2H), 7.45-7.36 (m, 2H); ^{13}C NMR (101 MHz, $CDCl_3$) δ 138.96, 137.56, 125.79, 125.65, 123.44, 121.97, 114.34, 111.83. These data are consistent with those reported in the literature [3].

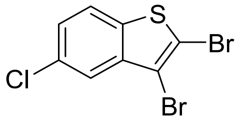

2,3-Dibromo-5-chlorobenzo[*b*]thiophene:

2,3-Dibromo-5-chlorobenzo[*b*]thiophene was synthesized from 5-chlorobenzo[*b*]thiophene (3 g, 17.8 mmol) following the similar procedure with the synthesis of 2,3-dibromo-benzo[*b*]thiophene. After purification by recrystallization from methanol/ethanol co-solvents (v/v = 2/1), 2,3-dibromo-5-chlorobenzo[*b*]thiophene (2.7 g, 8.3 mmol, 47%) was obtained as white solid. ^{1}H NMR (400 MHz,

$CDCl_3$) δ 7.72 (d, J = 2.1, 1H), 7.61 (d, J = 8.4, 1H), 7.34 (dd, J = 8.6, 1.9, 1H); ^{13}C NMR (101 MHz, $CDCl_3$) δ 138.70, 137.04, 132.22, 126.27, 123.10, 123.03, 116.25, 111.02. MS (EI): $[M]^+$ 326. These data are consistent with those reported in the literature [3].

2,3-Dibromo-6-octylbenzo[b]thiophene:

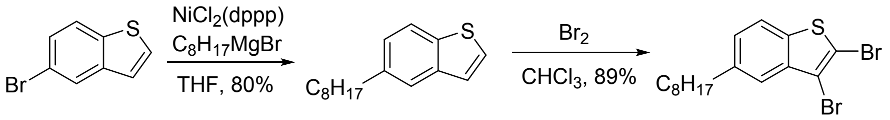

First step: preparation of 6-octylbenzo[*b*]thiophene:

6-Bromobenzo[*b*]thiophene (200 mg, 0.94 mmol) and $NiCl_2$(dppp) (49 mg, 0.1 mmol) was added into a 50 ml round-bottomed 2-necked flask containing 15 ml of distilled THF under N_2 atmosphere. $C_8H_{17}MgBr$ (0.7 ml, 1.9 mmol) was added to the tube slowly. It was stirred for 24 h at room temperature. The reaction mixture was quenched by NH_4Cl solution (1 mol/L) and then extracted for three times with ethyl acetate. The organic layer was washed with saturated brine, and then dried with $MgSO_4$. After the solvents were removed, the residue was purified by flash column chromatography on silica gel, affording 6-octylbenzo[*b*]thiophene (185 mg, 0.75 mmol, 80%) as colorless oily liquid. ^1H NMR (400 MHz, $CDCl_3$) δ 7.72 (d, J = 8.1 Hz, 1H), 7.68 (s, 1H), 7.35 (d, J = 5.5 Hz, 1H), 7.28 (d, J = 4.9 Hz, 1H), 7.19 (dd, J = 8.1, 1.5 Hz, 1H), 2.76 – 2.68 (m, 2H), 1.72 – 1.61 (m, 2H), 1.35 – 1.23 (m, 10H), 0.88 (t, J = 6.9 Hz, 3H). ^{13}C NMR (101 MHz, $CDCl_3$) δ 140.02, 139.29, 137.61, 125.43, 125.24, 123.58, 123.22, 121.70, 36.09, 31.91, 31.84, 29.49, 29.41, 29.27, 22.73, 14.09. MS (EI): $[M]^+$ 246. All the data are consistent with the literature reported [3].

Second step: preparation of 2,3-dibromo-6-octylbenzo[*b*]thiophene:

2,3-Dibromo-6-octylbenzo[*b*]thiophene (2.0 g, 8.1 mmol) was synthesized following the similar condition as the synthesis of 2,3-dibromobenzo[*b*]thiophene except that the temperature was not above 10 ^oC. 2,3-Dibromo-6-octylbenzo[*b*]thiophene (2.9 g, 7.2 mmol, 89%) was obtained as light yellow liquid. ^1H NMR (400 MHz, $CDCl_3$): δ 7.61 (d, J = 8.2 Hz, 1H), 7.48 (s, 1H), 7.22 (dd, J = 8.3, 1.3 Hz, 1H), 2.69 (t, J = 8.0 Hz, 2H), 1.63-1.70 (m, 2H), 1.26-1.30 (m, 10H), 0.87 (t, J = 6.9 Hz, 3H); ^{13}C NMR (101 MHz, $CDCl_3$): δ 141.15, 139.06, 135.57, 126.69, 123.06, 121.20, 112.88, 111.54, 36.07, 32.01, 31.69, 29.60, 29.46, 29.39, 22.82, 14.28. MS (EI): $[M]^+$ 404.

Synthesis of 5-(*tert*-butyl)-2-(4,4,5,5-tetramethyl-1,3,2-dioxaborolan-2-yl)phenol：

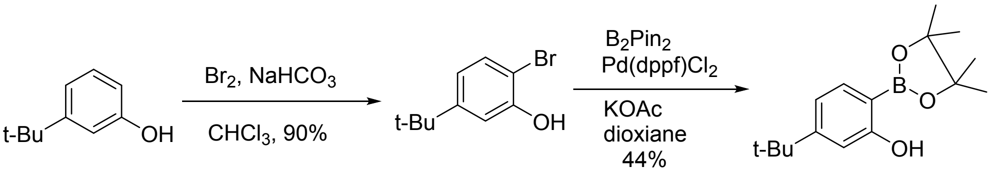

First step: preparation of 2-bromo-5-(*tert*-butyl)phenol:

3-(*Tert*-butyl)phenol (3 g, 20 mmol) was dissolved in 50 ml DCM and then Br_2 (1.04 ml, 20.3 mmol) was added into the solution under ice-water bath. After it was stirred for 7 h at room temperature, $NaHCO_3$ aqueous solution was added to neutralize the hydrobromic acid. The organic

layer was extracted with DCM and washed with saturated brine, and then dried with $MgSO_4$. After the solvent was removed, 2-bromo-5-(*tert*-butyl)phenol (4.1 g, 18 mmol, 90%) was obtained as yellow liquid. ^{1}H NMR (400 MHz, $CDCl_3$) δ 7.37 (d, J = 8.5 Hz, 1H), 7.08 (dd, J = 2.3, 1.1 Hz, 1H), 6.85 (dd, J = 8.5, 2.3 Hz, 1H), 5.56 (s, 1H), 1.30 (s, 9H). ^{13}C NMR (101 MHz, $CDCl_3$) δ 153.18, 151.83, 131.42, 119.28, 113.54, 106.93, 34.73, 31.26. These data are consistent with those reported in the literature [4].

Second step: preparation of 5-(*tert*-butyl)-2-(4,4,5,5-tetramethyl-1,3,2-dioxaborolan-2-yl)phenol：

2-Bromo-5-(*tert*-butyl)phenol (1.2 g, 5 mmol) and 4,4,4',4',5,5,5',5'-octamethyl-2,2'-bi-1,3,2-dioxa-borolane (B_2Pin_2) (1.5 g, 6 mmol) was dissolved in 50 ml dioxane-H_2O co-solvents (v/v = 4:1) in a 100 ml two-necked round bottom flask, then KOAc (0.8 g, 7.5 mmol) was added to the solution. After the solution was bubbled with N_2 for 20 min, $Pd(PPh_3)_2Cl_2$ (123 mg, 0.15 mmol) was added. Then the reaction was heated to 90 ^{o}C for 12 h. After the reaction mixture was cooled to room temperature, the organic layer was separated and dried with $MgSO_4$. It was filtered and the filtrate was evaporated under vacuum, the residue was purified by flash chromatography to afford the 5-(*tert*-butyl)-2-(4,4,5,5-tetramethyl-1,3,2-dioxaborolan-2-yl)phenol (0.54 g, 2.2 mmol, 44%) as white solid (mp 157.6 -158.8 °C). ^{1}H NMR (400 MHz, $CDCl_3$) δ 7.77 (s, 1H), 7.53 (d, J = 7.6 Hz, 1H), 6.94–6.92 (m, 2H), 1.34 (s, 12H), 1.29 (s, 9H). ^{13}C NMR (101 MHz, $CDCl_3$) δ 163.63, 158.03, 135.41, 117.09, 112.57, 84.39, 35.08, 31.14, 24.88. HRMS (ESI) calcd for $C_{16}H_{26}BO_3$ $[M+H]^+$ 277.1975, found 277.1971.

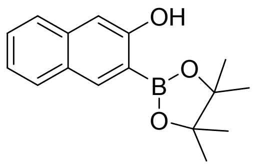

3-(4,4,5,5-Tetramethyl-1,3,2-dioxaborolan-2-yl)-2-naphthalenol (**2j**):

3-Iodo-2-naphthalenol (4 g, 14.8 mmol) and B_2Pin_2 (7.4 g, 29.6 mmol) was dissolved in 50 ml dioxane-H_2O co-solvents (v/v = 4:1) in a 100 ml two-necked round bottom flask, then KOAc (3.3 g, 29.6 mmol) was added to the solution. After the solution was bubbled with N_2 for 20 min, $Pd(PPh_3)_2Cl_2$ (855 mg, 0.74 mmol, 5 mol %) was added. Then the reaction mixture was heated to 90 ^{o}C for 12 h. After the reaction mixture was cooled to room temperature, the organic layer was separated and dried with $MgSO_4$. It was filtered and the filtrate was evaporated under vacuum, the residue was purified by flash chromatography to afford 4-(4,4,5,5-tetramethyl-1,3,2-dioxaborolan-2-yl)-2-naphthalenol (2.1 g, 7.8 mmol, 53%) as light-yellow solid (mp 56.3 - 57.1 °C). ^{1}H NMR (400 MHz, DMSO-D_6) δ 8.33 (s, 1H), 7.25 (s, 1H), 6.97 (d, J = 8.2 Hz, 1H), 6.79 (d, J = 7.8 Hz, 1H), 6.57-6.53 (m, 1H), 6.40-6.36 (m, 1H), 6.25 (s, 1H), 0.42 (s, 12H); ^{13}C NMR (101 MHz, DMSO-D_6) δ 158.87, 138.01, 136.29, 128.30, 127.47, 127.30, 125.75, 122.86, 118.18, 108.48, 83.58, 24.70. HRMS (ESI) calcd for $C_{16}H_{19}BO_3$ $[M-H]^-$ 269.1354, found 269.1352.

General procedure for screening of reaction conditions (entries 1-20 in Table 1):

Compound **1a** (0.48 mmol), **2a** (0.4 mmol) and base (1.2 mmol) were added into 25 ml Schlenk tube. Then *tert*-butanol (5 ml, containing 5 mol % 18-crown-6 for some entries) was added under nitrogen atmosphere. After the solution was bubbled for 10 min with nitrogen, $Pd(PPh_3)_4$ (0.5-5 mol %) was added to the reaction system. After the reaction was stirred at 90 °C for 24 h, copper catalyst (2-5 mol %) were added to the mixture (200 mg activated 3 Å molecular sieves was added as water adsorbent for some entries), and then it was stirred for another 4 h. TCE (100 μl) was added as an internal standard for GC analysis. About 2 ml ethyl acetate and 2 ml petroleum ether were added for extraction. The GC sample was prepared by dilution of 0.1 ml the above organic layer with 2 mL ethyl acetate. GC yield was determined using standard curve with TCE as an internal standard (see Figure S1). The product has the same retention time with authentic sample which is synthesized by conventional method. [4]

General procedure for synthesis of **3a-3f**:

Compound **1** (0.48 mmol), **2** (0.4 mmol) and K_3PO_4•$3H_2O$ (319 mg, 1.2 mmol) were added to 25 ml Schlenk tube. *Tert*-butanol (5 ml) containing 5 mol % 18-crown-6 was added under nitrogen atmosphere. After the solution was bubbled for 10 min with nitrogen, Pd $(PPh_3)_4$ (2 mol %) was added to the reaction system. After the reaction was stirred at 90 °C for 24 h, 200 mg activated 3Å molecular sieves and CuTc (2 mol %) were added to the mixture, and then it was stirred for another 4 h. After the removal of the solvents under reduced pressure, the residue was purified by flash chromatography with petroleum ether as an eluent to afford target molecules.

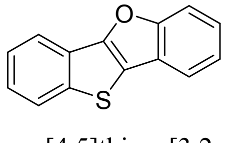

Benzo[4,5]thieno[3,2-*b*] benzofuran (**3a**): white solid (71 mg, 70% yield). ^{1}H NMR (400 MHz, $CDCl_3$): δ 8.01 (d, J = 7.8 Hz, 1H), 7.88 (d, J = 8.1 Hz, 1H), 7.73 (d, J = 6.9 Hz, 1H), 7.65 (d, J = 7.8 Hz, 1H), 7.50-7.32 (m, 4H); ^{13}C NMR (101 MHz, $CDCl_3$) δ 158.89, 153.10, 142.10, 125.24, 125.06, 125.03, 124.48, 124.16, 123.42, 119.82, 119.71, 118.69, 112.68. These data are consistent with those reported in the literature [3].

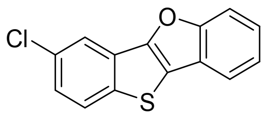

3-Chlorobenzo[4,5]thieno[3,2-*b*]benzofuran (**3b**): white solid (52 mg, 60% yield). ^{1}H NMR (400 MHz, $CDCl_3$) δ 7.95 (d, J = 2.1 Hz, 1H), 7.76 (d, J = 8.6 Hz, 1H), 7.71 (dd, J = 7.7, 1.2 Hz, 1H), 7.63 (d, J = 8.2 Hz, 1H), 7.43 – 7.29 (m, 3H); ^{13}C NMR (101 MHz, $CDCl_3$) δ 158.98, 151.91,

140.02, 131.41, 126.19, 125.58, 125.39, 125.28, 123.84, 123.61, 120.55, 119.89, 119.55, 112.83. All the data are consistent with the literature reported [3].

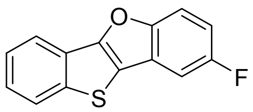

8-Fluorobenzo[4,5]thieno[3,2-*b*]benzofuran (**3c**): white solid (45 mg, 44% yield). ^{1}H NMR (400 MHz, $CDCl_3$) δ 8.01 (d, J = 8.1 Hz, 1H), 7.89 (d, J = 8.1 Hz, 1H), 7.58 (dd, J = 9.0, 4.1 Hz, 1H), 7.54 – 7.45 (m, 1H), 7.45 – 7.37 (m, 2H), 7.10 (td, J = 9.0, 2.7 Hz, 1H); ^{13}C NMR (101MHz, $CDCl_3$) δ 159.33 (d, J = 238 Hz), 154.80 (d, J = 31 Hz), 142.25, 125.43, 125.14, 124.98, 124.83, 124.72, 124.47, 119.95, 118.39, 113.21 (d, J = 10 Hz), 112.38 (d, J = 26 Hz), 105.64 (d, J = 26 Hz). All the data are consistent with the literature reported [3].

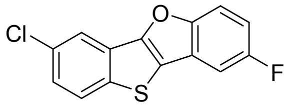

3-Chloro-8-fluorobenzo[4,5]thieno[3,2-*b*]benzofuran (**3d**): white solid (34 mg, 38% yield). ^{1}H NMR (400 MHz, $CDCl_3$) δ 7.98 (d, J = 2.3 Hz, 1H), 7.80 (d, J = 8.7 Hz, 1H), 7.59 (dd, J = 9.1, 4.1 Hz, 1H), 7.42-7.36 (m, 2H), 7.13 (td, J = 9.1, 2.7 Hz, 1H); ^{13}C NMR (101 MHz, $CDCl_3$) δ 159.38 (d, J = 243 Hz), 155.03, 153.40, 140.13, 131.52, 125.92, 125.71, 125.37, 124.50, 124.39, 119.67, 113.43 (d, J = 10 Hz), 113.01 (d, J = 26 Hz), 105.82 (d, J = 26 Hz). All the data are consistent with the literature reported [3].

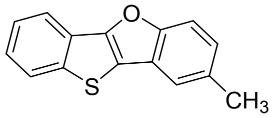

8-Methylbenzo[4,5]thieno[3,2-*b*]benzofuran (**3e**): white solid (35 mg, 36% yield), mp 111.1-112.4 °C. ^{1}H NMR (400MHz, $CDCl_3$) δ 7.99 (d, J = 8.2 Hz, 1H), 7.87 (d, J = 8.2 Hz, 1H), 7.51-7.53 (m, 2H), 7.46 (td, J = 7.5, 0.9 Hz, 1H), 7.35-7.39 (m, 1H), 7.17-7.19 (m, 1H), 2.50 (s, 3H); ^{13}C NMR (101 MHz, $CDCl_3$) δ 157.29, 153.22, 142.00, 133.02, 126.19, 125.32, 125.01, 124.89, 124.46, 124.10, 119.73, 119.66, 118.46, 112.13, 21.54. HRMS (EI) calcd for $C_{15}H_{10}OS$ $[M]^+$ 238.0452, found 238.0443.

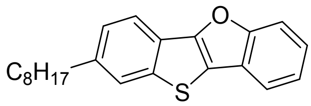

8-Octylbenzo[4,5]thieno[3,2-*b*]benzofuran (**3f**): white solid (74 mg, 61% yield), mp 57.4 - 58.7 °C.

^{1}H NMR (400 MHz, CDCl$_3$) δ 7.91 (d, J = 8.2 Hz, 1H), 7.63-7.72 (m, 3H), 7.29-7.39 (m, 3H), 2.76 (t, J = 7.5 Hz, 2H), 1.66-1.73 (m, 2H), 1.25-1.42 (m, 10H), 0.88 (t, J = 6.9 Hz, 3H); ^{13}C NMR (101 MHz, CDCl$_3$) δ 158.74, 153.18, 142.49, 140.44, 126.10, 124.67, 124.35, 123.81, 123.34, 123.22, 119.49, 119.47, 117.68, 112.58, 36.29, 32.02, 31.83, 29.62, 29.45, 29.39, 22.81, 14.26. HRMS (EI) calcd for $C_{22}H_{24}OS$ [M]$^+$ 336.1548, found 336.1544.

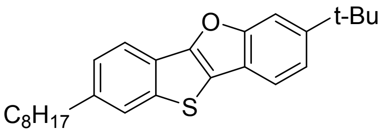

2-Octyl-7-*tert*-butylbenzo[4,5]thieno[3,2-*b*]benzofuran (**3g**):

The title compound was prepared from was prepared from 2,3-dibromo-6-octylbenzo[*b*]thiophene (194 mg, 0.48 mmol) and 5-(*tert*-butyl)-2-(4,4,5,5-tetramethyl-1,3,2-dioxaborolan-2-yl)phenol (110 mg, 0.4 mmol) as colorless liquid (63 mg, 40% yield). ^{1}H NMR (400 MHz, CDCl$_3$) δ 7.88 (d, J = 8.2 Hz, 1H), 7.70-7.65 (m, 2H), 7.62 (d, J = 8.2 Hz, 1H), 7.40 (dd, J = 8.2, 1.8 Hz, 1H), 7.29 (dd, J = 8.2, 1.4 Hz, 1H), 2.75 (t, J = 7.8 Hz, 2H), 1.65-1.73 (m, 2H), 1.42 (s, 9H), 1.27-1.39 (m, 10H), 0.88 (t, J = 6.9 Hz, 3H); ^{13}C NMR (101 MHz, CDCl$_3$) δ 159.19, 153.02, 148.81, 142.25, 140.10, 126.02, 123.80, 123.38, 121.71, 121.05, 119.26, 118.75, 117.58, 109.40, 36.29, 35.23, 32.02, 31.87, 31.78, 29.63, 29.47, 29.40, 22.81, 14.27. HRMS (EI) calcd for $C_{26}H_{32}OS$ [M]$^+$ 392.2174, found 392.2165.

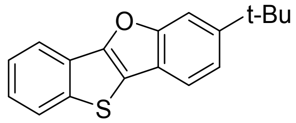

7-(*Tert*-butyl)benzo[4,5]thieno[3,2-*b*]benzofuran (**3h**):

The title compound was prepared from 2,3-dibromobenzo[*b*]thiophene (140 mg, 0.48 mmol) and 5-(*tert*-butyl)-2-(4,4,5,5-tetramethyl-1,3,2-dioxaborolan-2-yl)phenol (110 mg, 0.4 mmol) as white solid (56 mg, 47% yield). ^{1}H NMR (400 MHz, CDCl$_3$) δ 7.98 (d, J = 8.0 Hz, 1H), 7.87 (d, J = 8.0 Hz, 1H), 7.67 (d, J = 1.2 Hz, 1H), 7.64 (d, J = 8.0 Hz, 1H), 7.34–7.48 (m, 3H), 1.42 (s, 9H); ^{13}C NMR (100 MHz, CDCl$_3$) δ 159.33, 152.91, 149.16, 141.85, 125.36, 124.90, 124.63, 124.38, 121.49, 121.07, 119.53, 118.89, 118.58, 109.41, 35.19, 31.70. All the data are consistent with the literature reported [3].

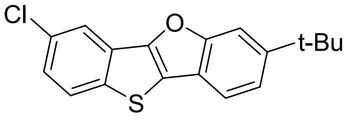

3-Chloro-7-*tert*-butylbenzo[4,5]thieno[3,2-*b*]benzofuran (**3i**):

The title compound was prepared from 2,3-dibromo-5-chlorobenzo[*b*]thiophene (156 mg, 0.48 mmol) and 5-(*tert*-butyl)-2-(4,4,5,5-tetramethyl-1,3,2-dioxaborolan-2-yl)phenol (110 mg, 0.4 mmol): white

solid (41 mg, 32% yield), mp 64.6 - 65.3 °C. ^{1}H NMR (400 MHz, $CDCl_3$) δ 7.96 (d, J = 2.0 Hz, 1H), 7.78 (d, J = 8.5 Hz, 1H), 7.68-7.65 (m, 2H), 7.43 (dd, J = 8.3, 1.6 Hz, 1H), 7.33 (dd, J = 8.6, 1.9 Hz, 1H), 1.42 (s, 9H); ^{13}C NMR (101 MHz, $CDCl_3$) δ 160.42, 158.03, 154.90, 153.27, 140.00, 131.39, 125.78, 125.58, 125.25, 119.54, 113.35, 113.26, 105.63, 105.57, 29.70, 14.07. HRMS (EI) calcd for $C_{18}H_{15}ClOS$ $[M]^+$ 314.0532, found 314.0529.

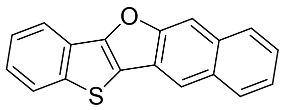

Benzo[4,5]thieno[3,2-*b*]naphthalenefuran (**3j**):

The title compound was prepared from 2,3-dibromobenzo[*b*]thiophene (140 mg, 0.48 mmol) and 3-(4,4,5,5-Tetramethyl-1,3,2-dioxaborolan-2-yl)-2-naphthalenol (108 mg, 0.4 mmol) as white solid (39 mg, 35% yield), mp 244.7 - 245.9 °C. ^{1}H NMR (400 MHz, $CDCl_3$) δ 8.16 (s, 1H), 8.02-8.05 (m, 2H), 7.96-8.00 (m, 2H), 7.91 (d, J = 8.2 Hz, 1H), 7.46-7.52 (m, 3H), 7.40-7.44 (m, 1H); ^{13}C NMR (101 MHz, $CDCl_3$) δ 156.93, 154.02, 141.64, 130.47, 129.57, 127.13, 127.00, 124.48, 124.41, 124.15, 123.75, 123.49, 119.18, 116.99, 116.50, 107.37. HRMS (EI) calcd for $C_{18}H_{10}OS$ $[M]^+$ 274.0452, found 274.0448.

4. Measurement of photophysical data

UV-Visible spectra of **3a, 3c, 3d, 3e** and **3j** were obtained on Shimadzu UV-1800ENG240V, SOFT spectrometer. Ca. 6×10^{-5} M of sample solutions in ethanol were used for ambient temperature measurement. As shown in Figure 1, the absorption spectra of these compounds exhibit fine structures, and the maximum absorption peaks at 318, 320, 319, 319 and 355 nm.

Fluorescence spectra of **3a, 3c, 3d, 3e** and **3j** were obtained on Shimadzu-RF-5301pc spectrometer with Ca. 2×10^{-7} M of sample solutions in ethanol were used for ambient temperature measurement. Using the maximum absorption wavelength as the excitation wavelength for fluorescence measurement, we found that the maximum emission peaks of these compounds are located at 339, 342, 347, 340, 378 nm, respectively.

9,10-Diphenylanthracene (Φ_f = 0.95 in ethanol) is used as the reference compound for the measurement of fluorescence quantum yield. Ca. 5×10^{-6} M of sample and reference solutions in ethanol were used to make sure that the absorption intensity (A_u and A_s) is below 0.1. Through software processing, we integrate the fluorescence spectra and get the integral area labeled F_u (Fluorescence intensity of the compounds to be measured). Similarly, the fluorescence spectra of

9,10-diphenylanthracene and the integral area labeled F_s (Fluorescence intensity of reference material) were measured. Φ_u (Fluorescence quantum yield of the compounds to be measured) can be obtained from formula $A_u \cdot \Phi_u \cdot F_s = A_s \cdot \Phi_s \cdot F_u$.

5. ^{1}H and ^{13}C NMR spectra and HRMS spectra

^{1}H NMR spectrum of 2,3-dibromobenzo[*b*]thiophene

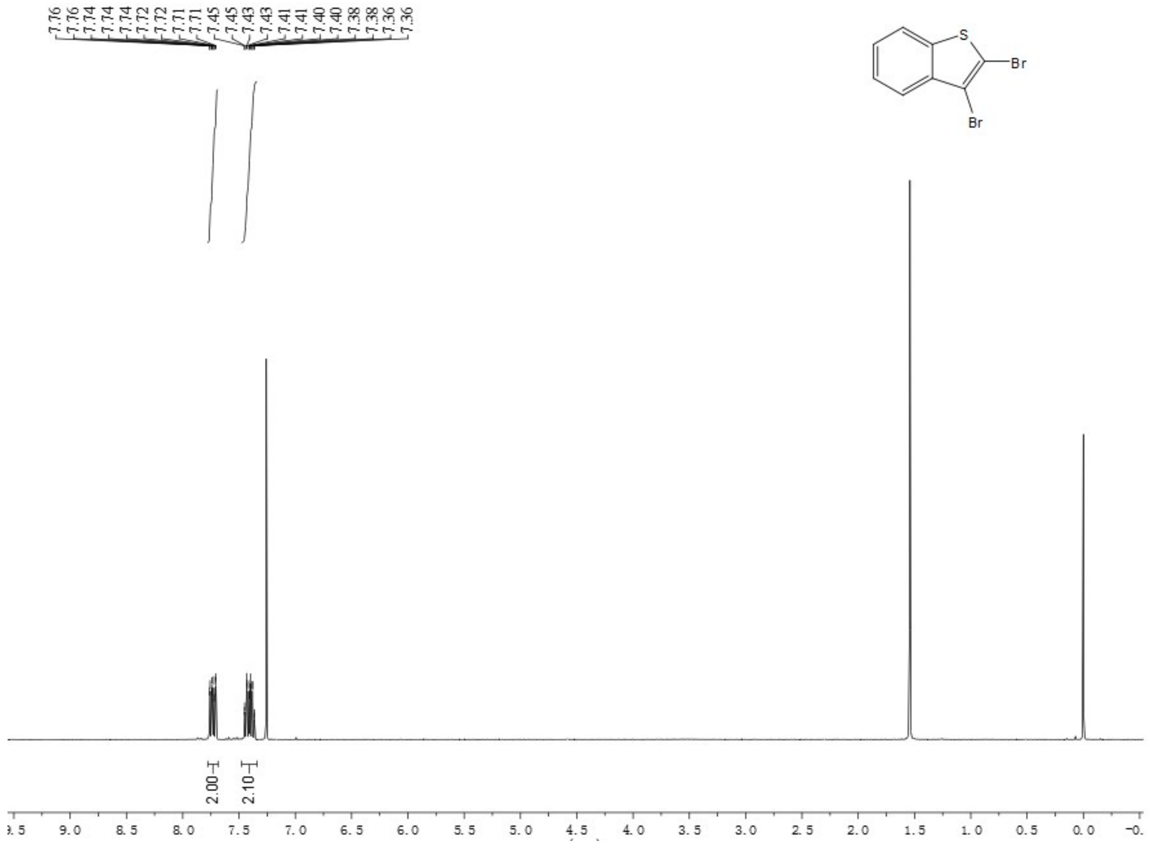

^{13}C NMR spectrum of 2,3-dibromobenzo[*b*]thiophene

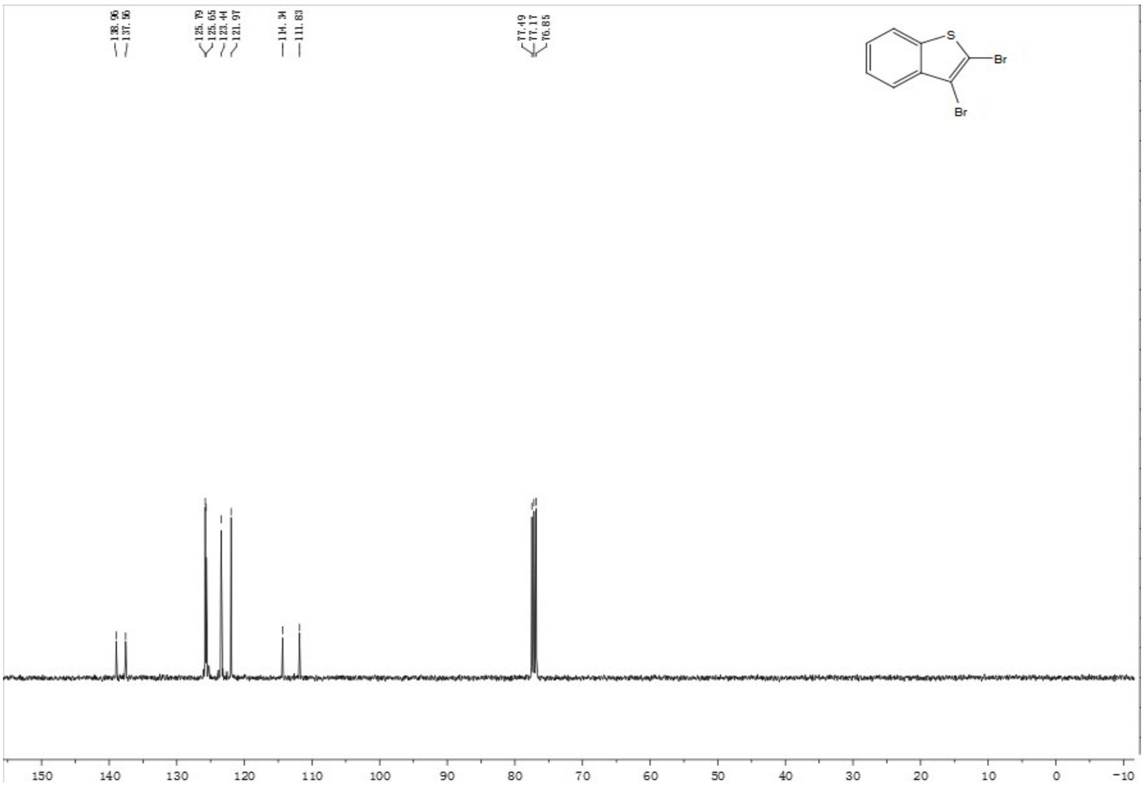

^{1}H NMR spectrum of 2,3-dibromo-5-chlorobenzo[*b*]thiophene

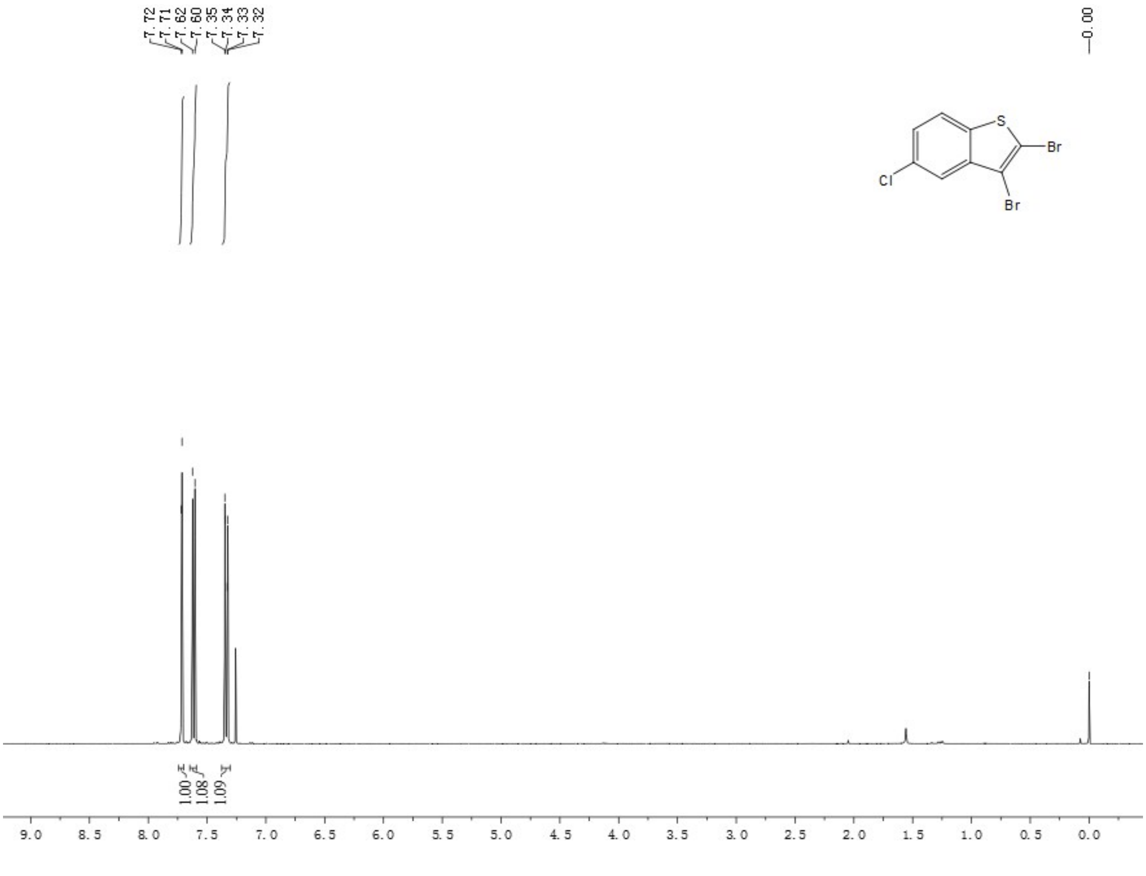

^{13}C NMR spectrum of 2,3-dibromo-5-chlorobenzo[*b*]thiophene

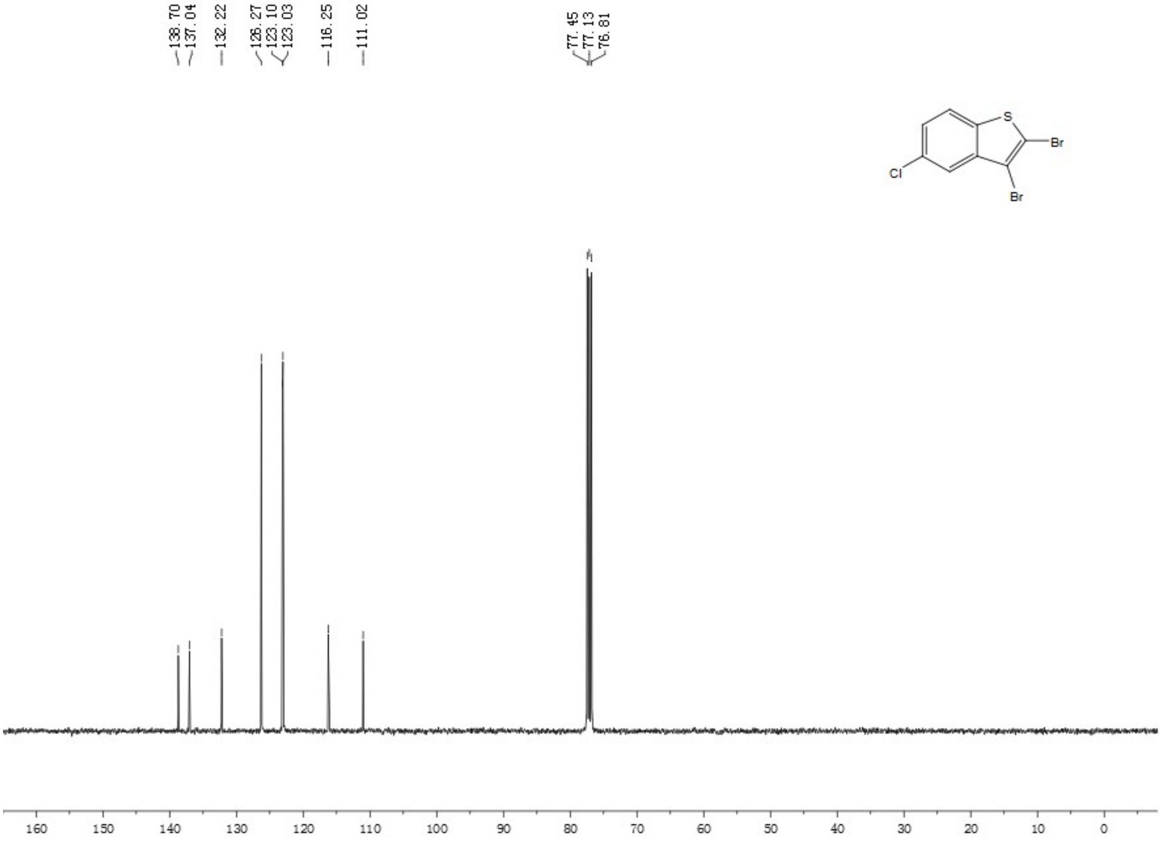

^{1}H NMR spectrum of 6-octylbenzo[*b*]thiophene

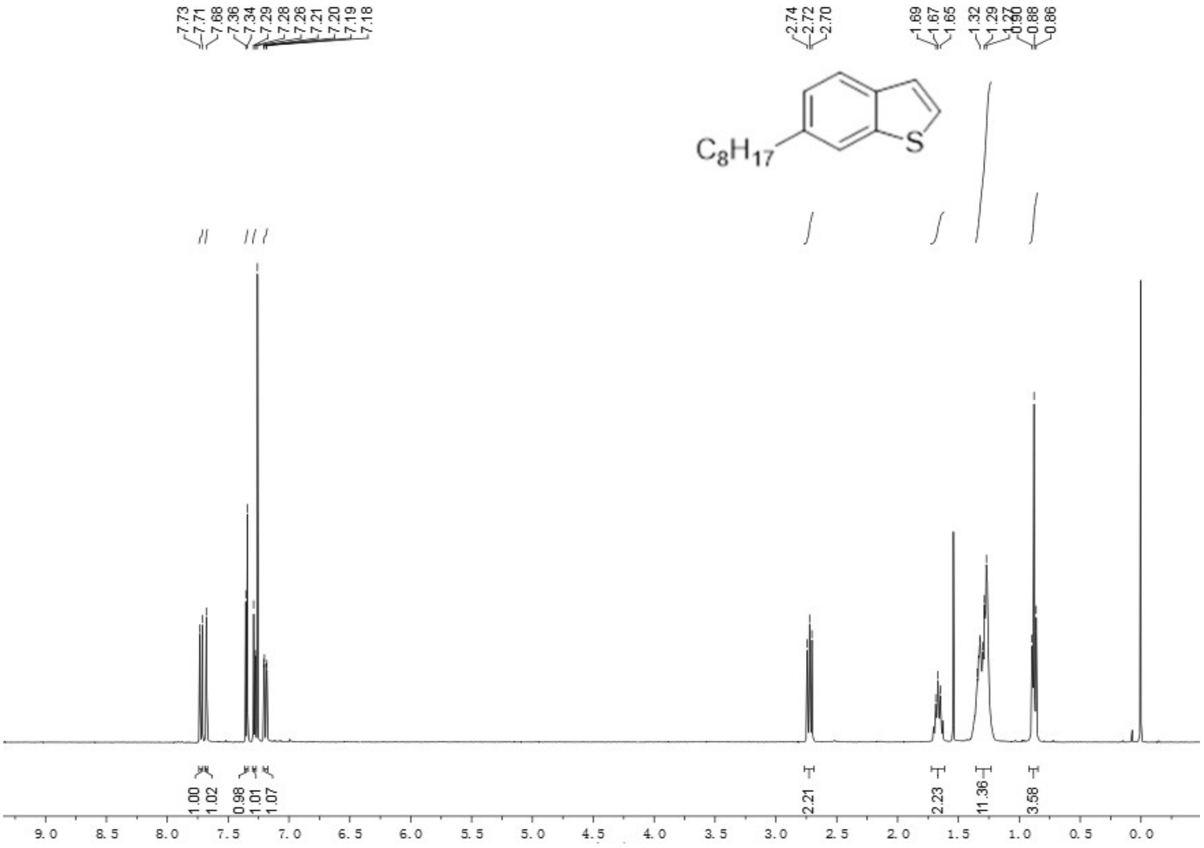

^{1}H NMR spectrum of 2,3-dibromo-6-octylbenzo[*b*]thiophene

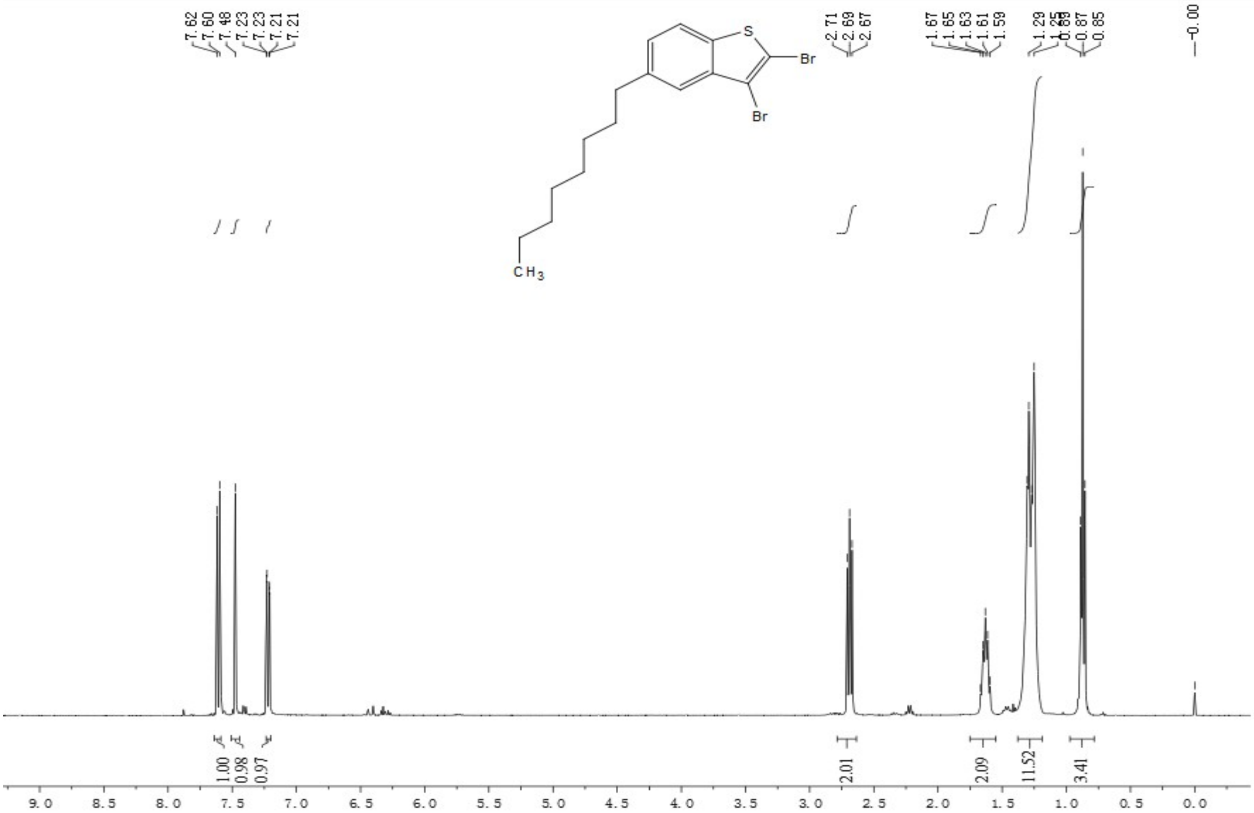

^{13}C NMR spectrum of 2,3-dibromo-6-octylbenzo[*b*]thiophene

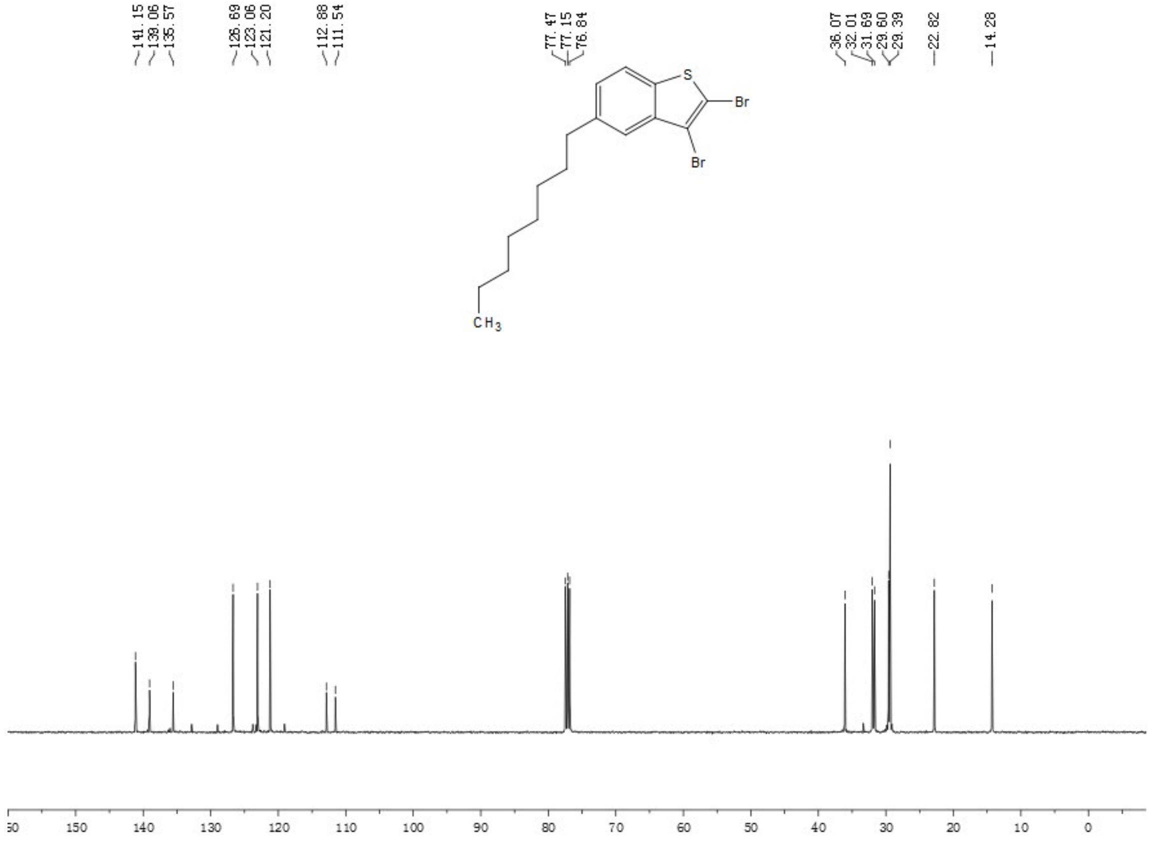

^{1}H NMR spectrum of 2-bromo-5-(*tert*-butyl)phenol

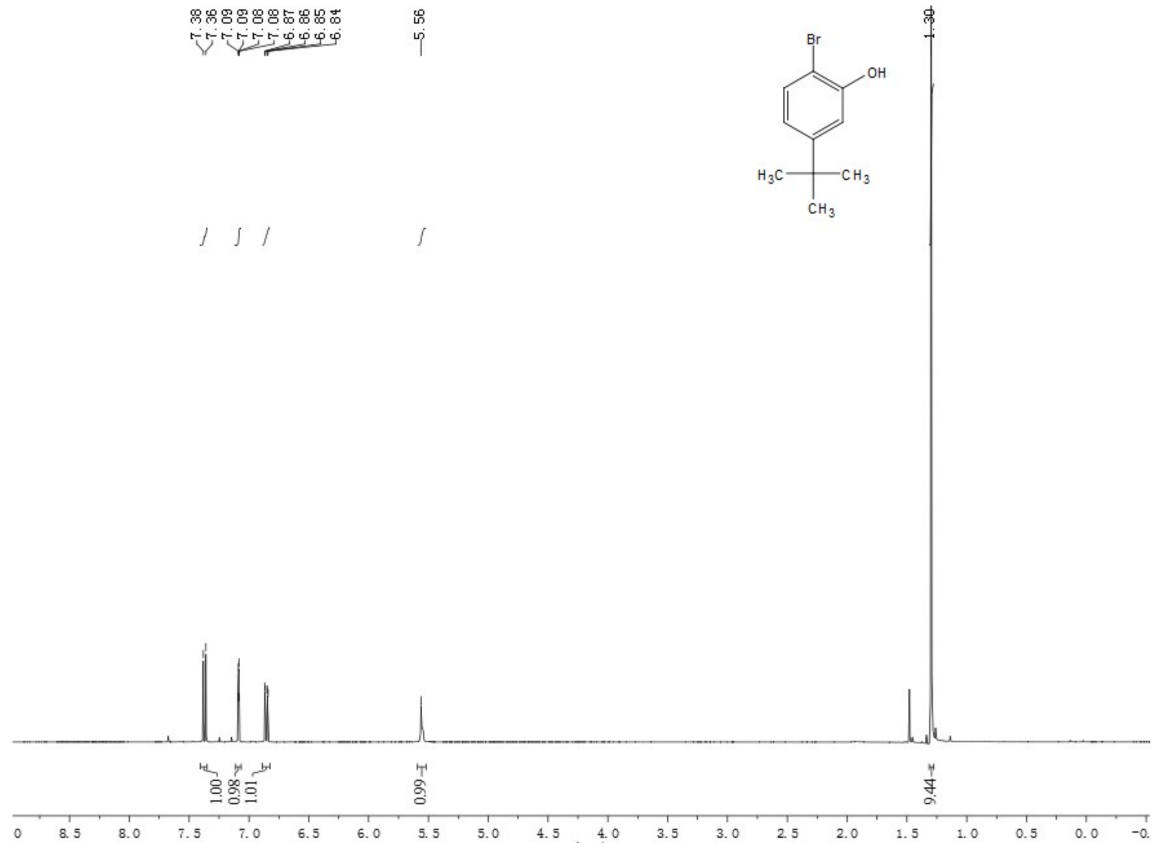

^{13}C NMR spectrum of 2-bromo-5-(*tert*-butyl)phenol

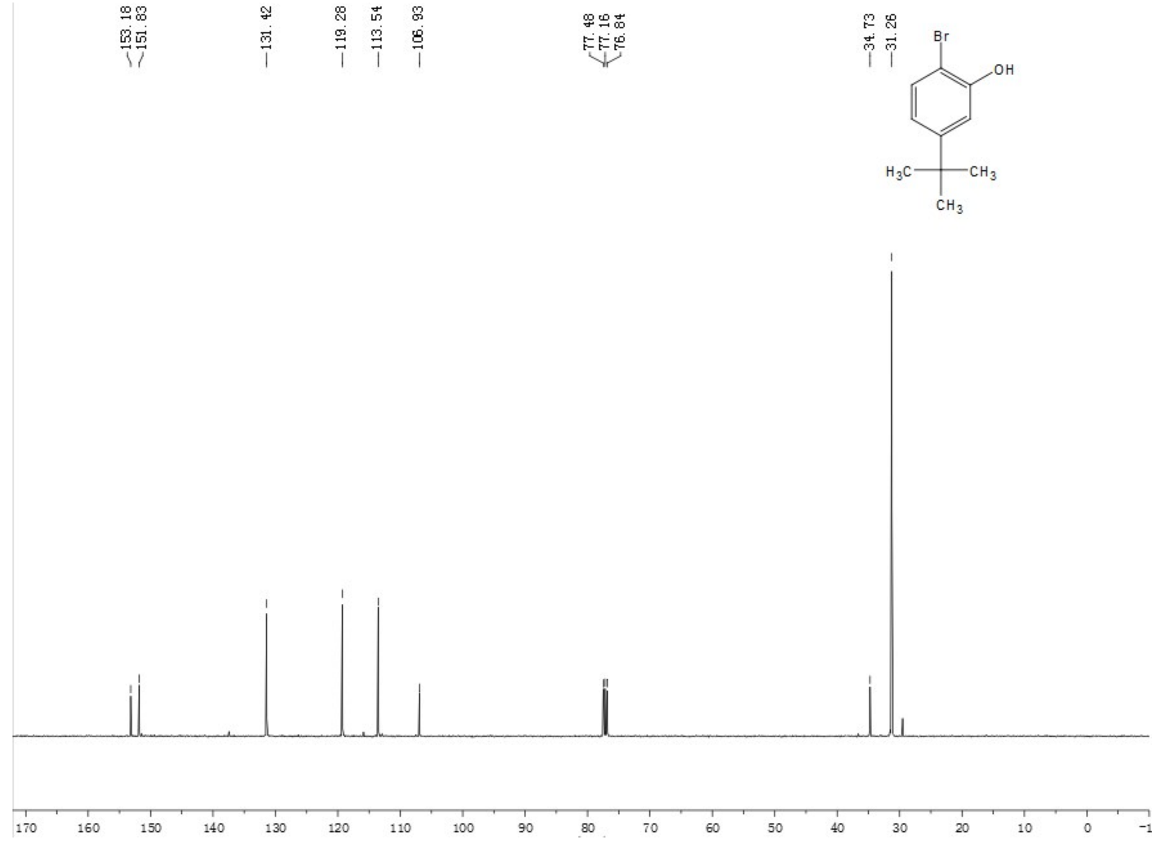

^{1}H NMR spectrum of 5-(*tert*-butyl)-2-(4,4,5,5-tetramethyl-1,3,2-dioxaborolan-2-yl)phenol

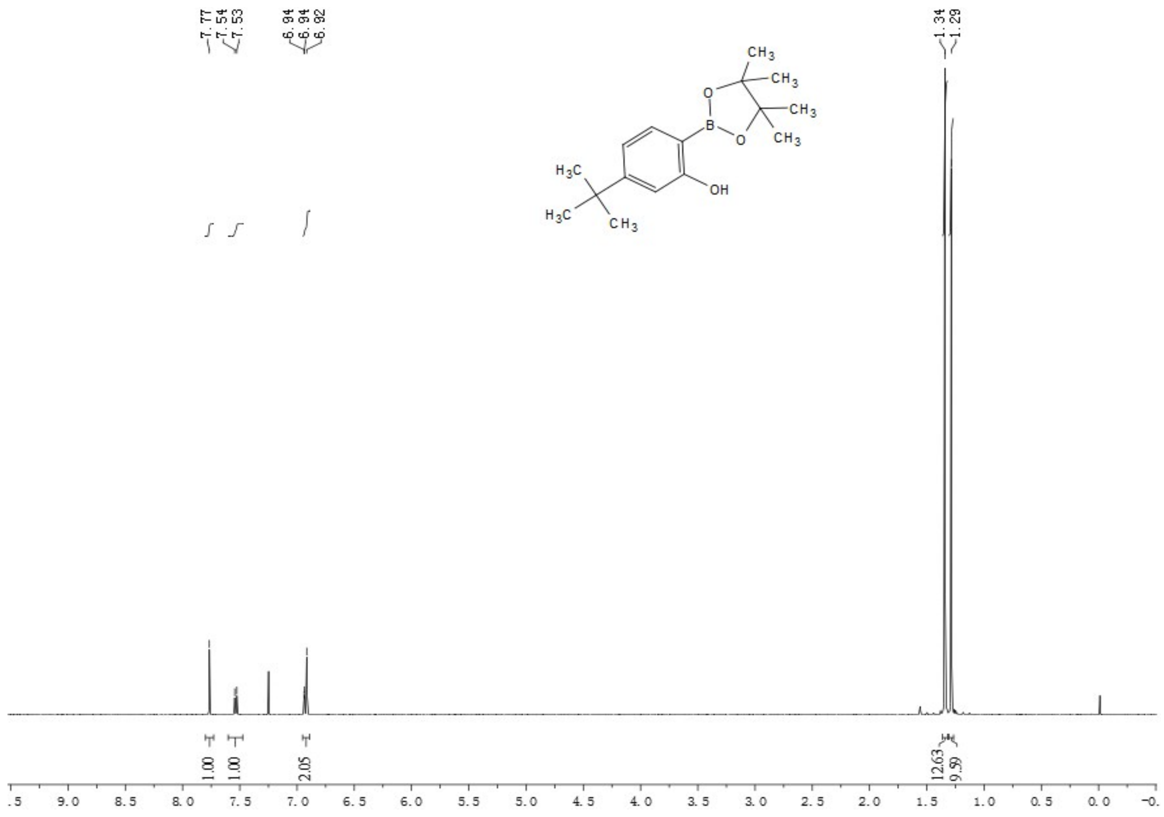

^{13}C NMR spectrum of 5-(*tert*-butyl)-2-(4,4,5,5-tetramethyl-1,3,2-dioxaborolan-2-yl)phenol

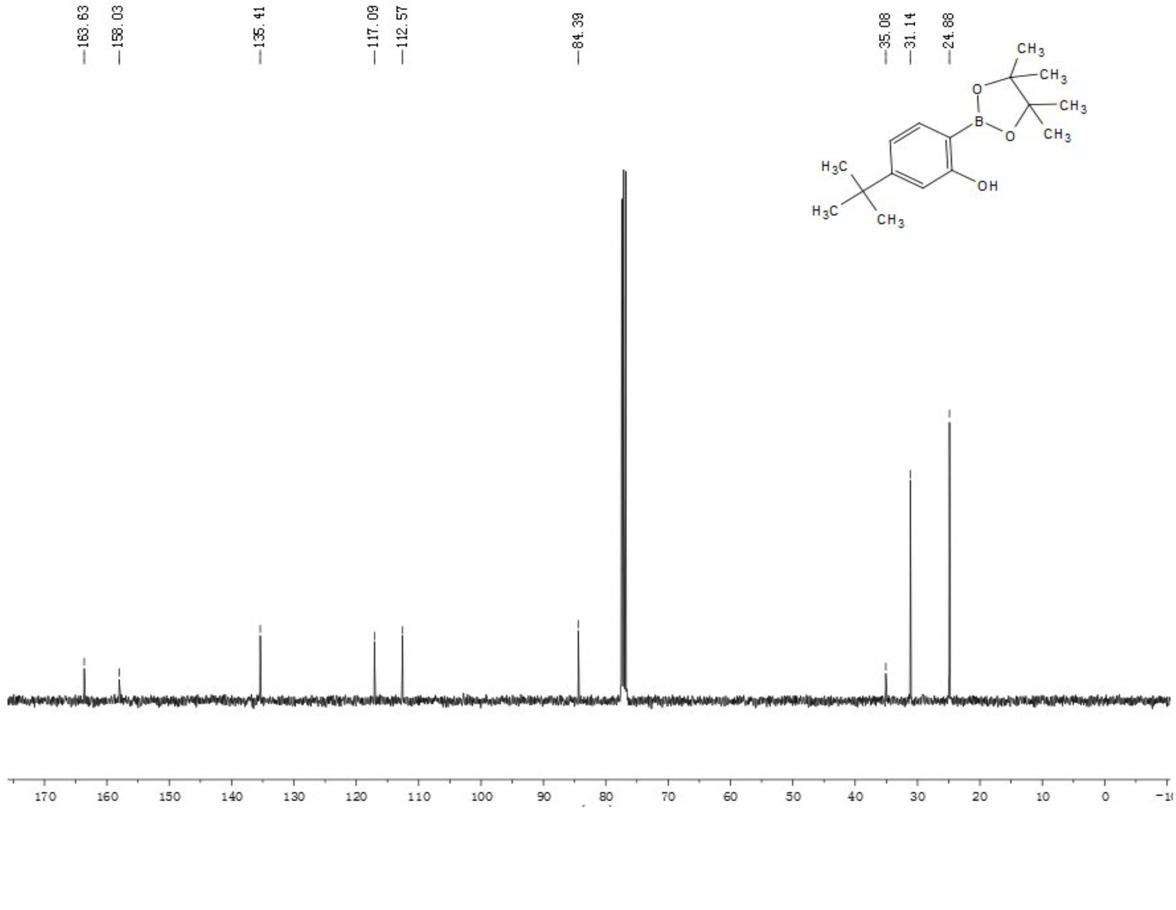

HR-MS spectrum of 5-(*tert*-butyl)-2-(4,4,5,5-tetramethyl-1,3,2-dioxaborolan-2-yl)phenol:

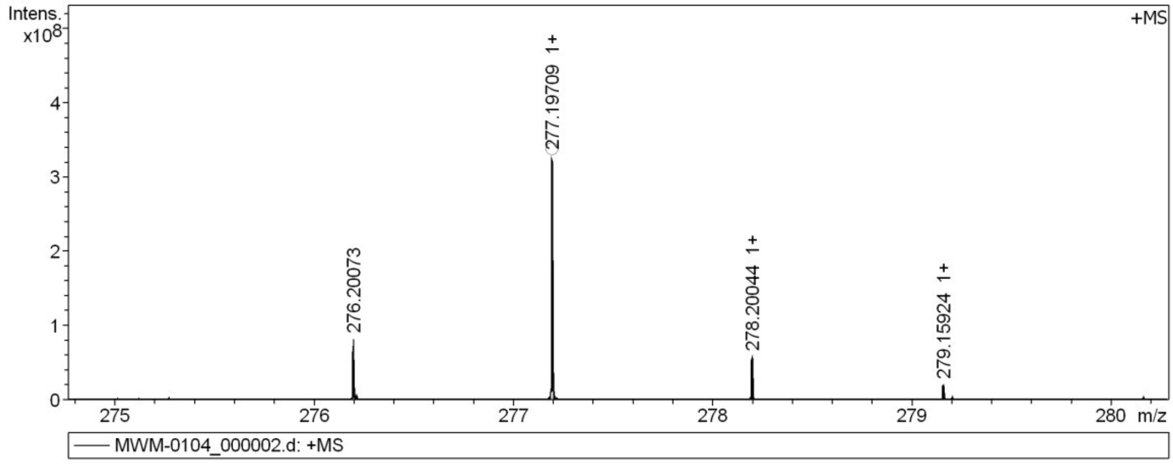

Meas. m/z	#	Ion Formula	Score	m/z	err [ppm]	Mean err [ppm]	mSigma	rdb	e⁻ Conf	N-Rule
277.197087	1	C16H26BO3	100.00	277.197248	-0.6	0.3	7.4	4.5	even	ok
299.179055	1	C16H25BNaO3	100.00	299.179192	0.5	0.2	4.9	4.5	even	ok
315.152971	1	C16H25BKO3	100.00	315.153130	0.5	0.6	9.5	4.5	even	ok

^{1}H NMR spectrum of **2j**

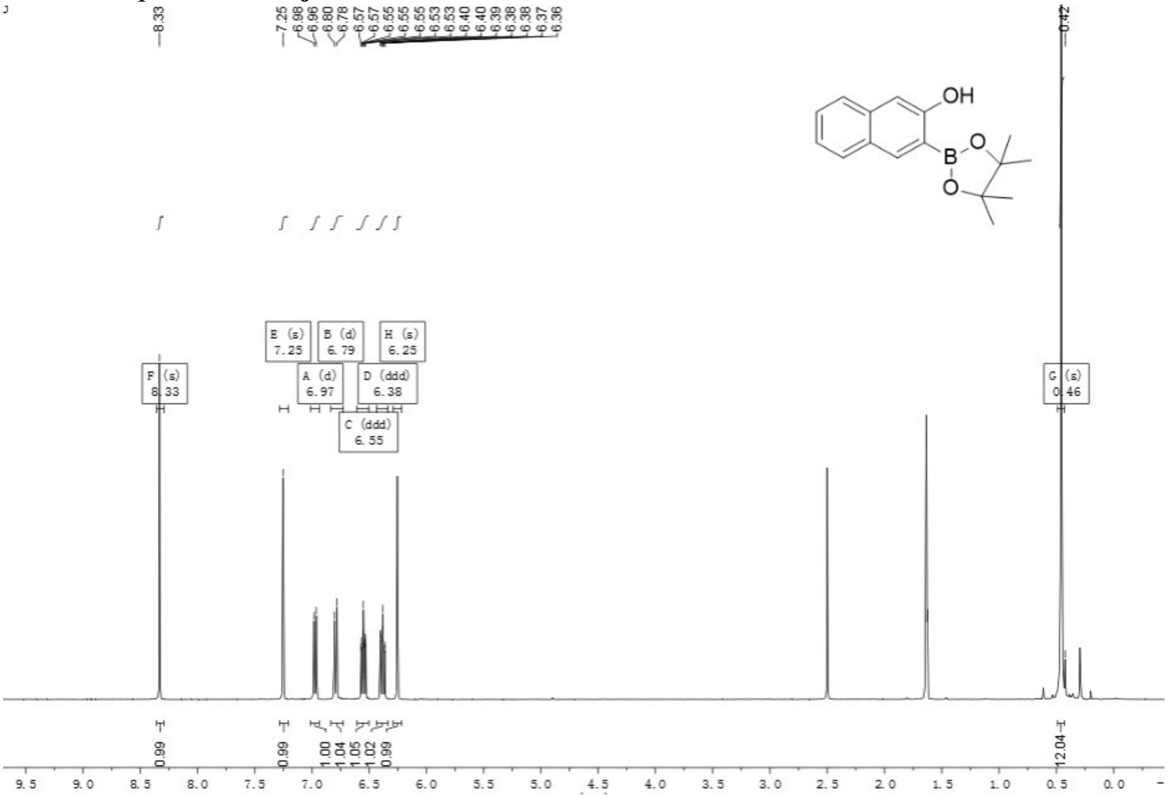

^{13}C NMR spectrum of **2j**

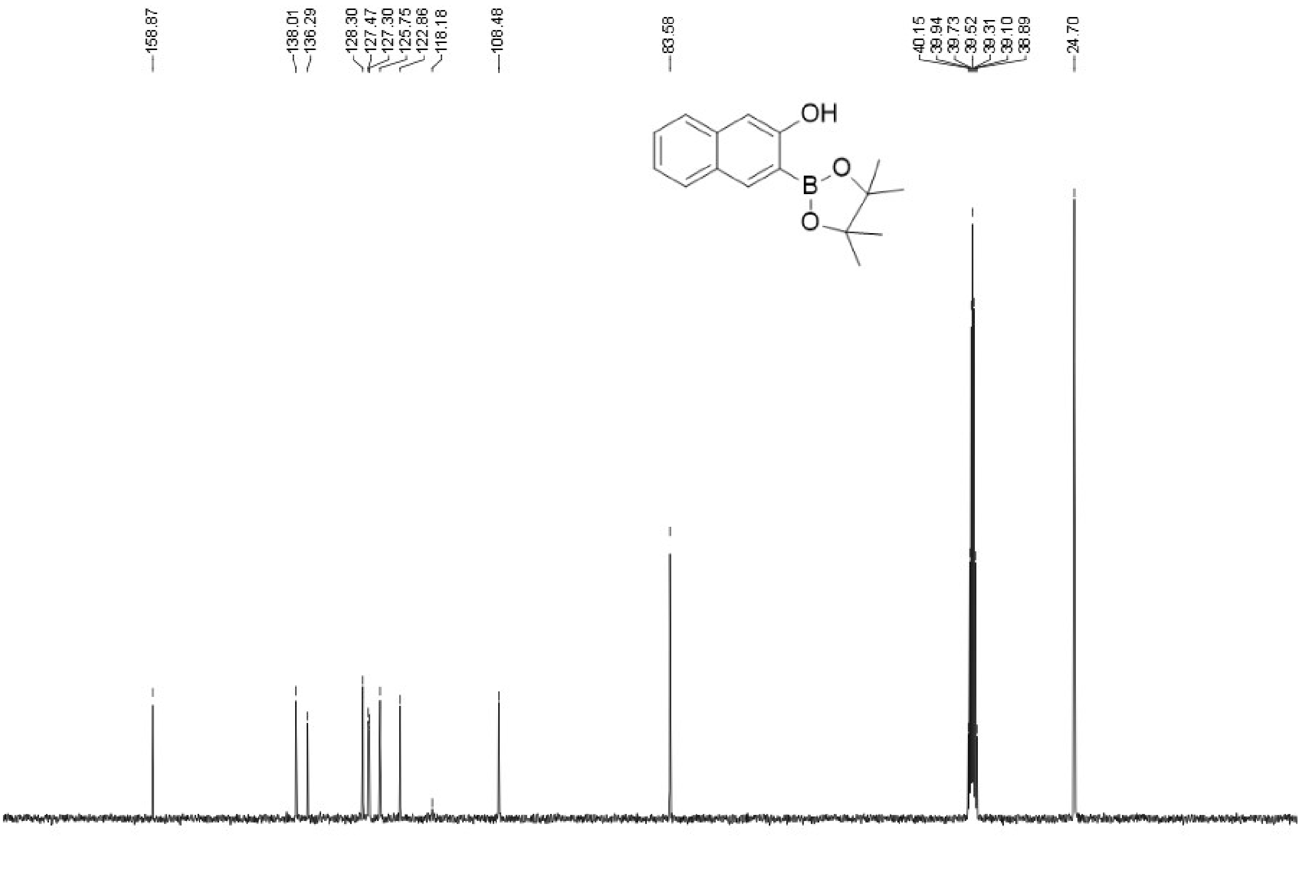

HRMS spectrum of **2j**

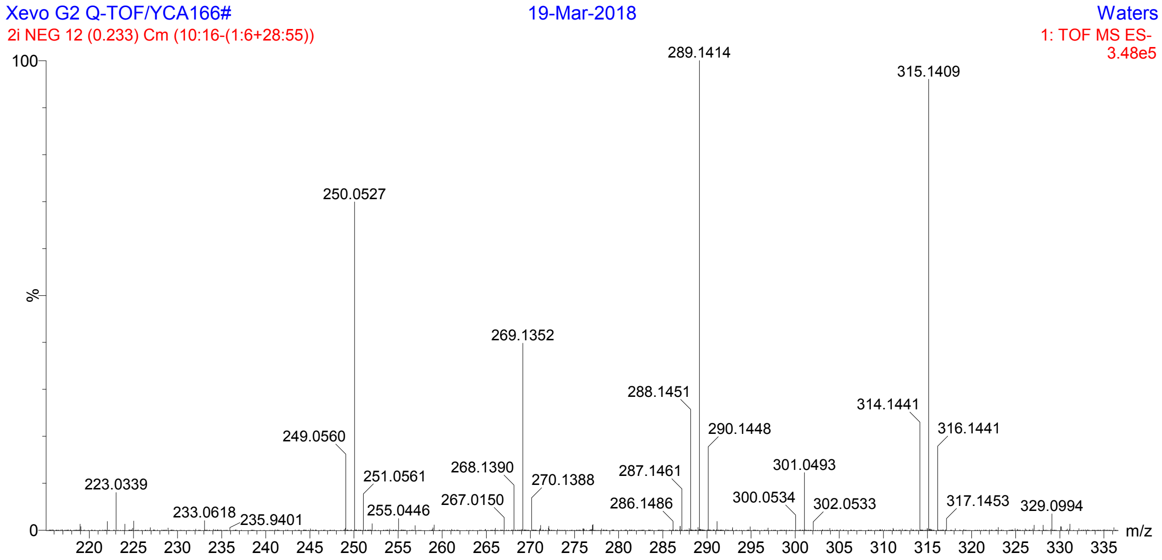

^{1}H NMR spectrum of **3a**

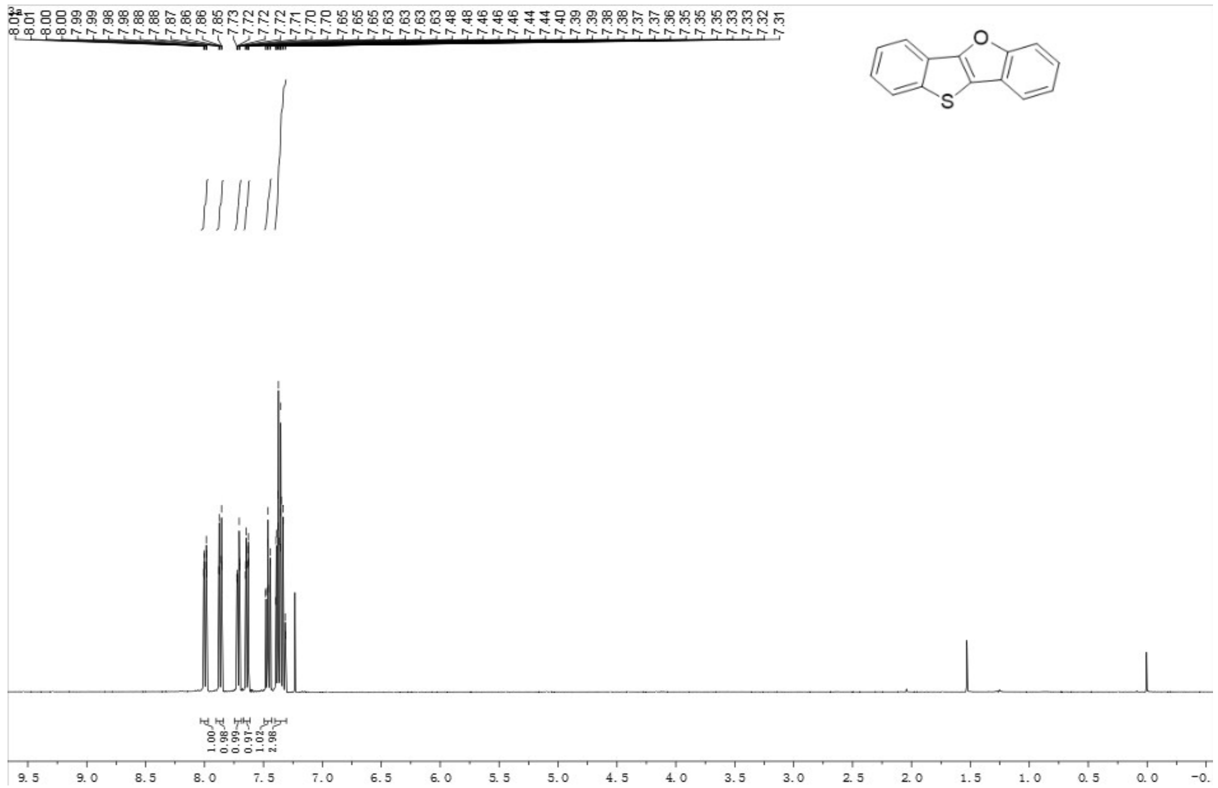

^{13}C NMR spectrum of **3a**

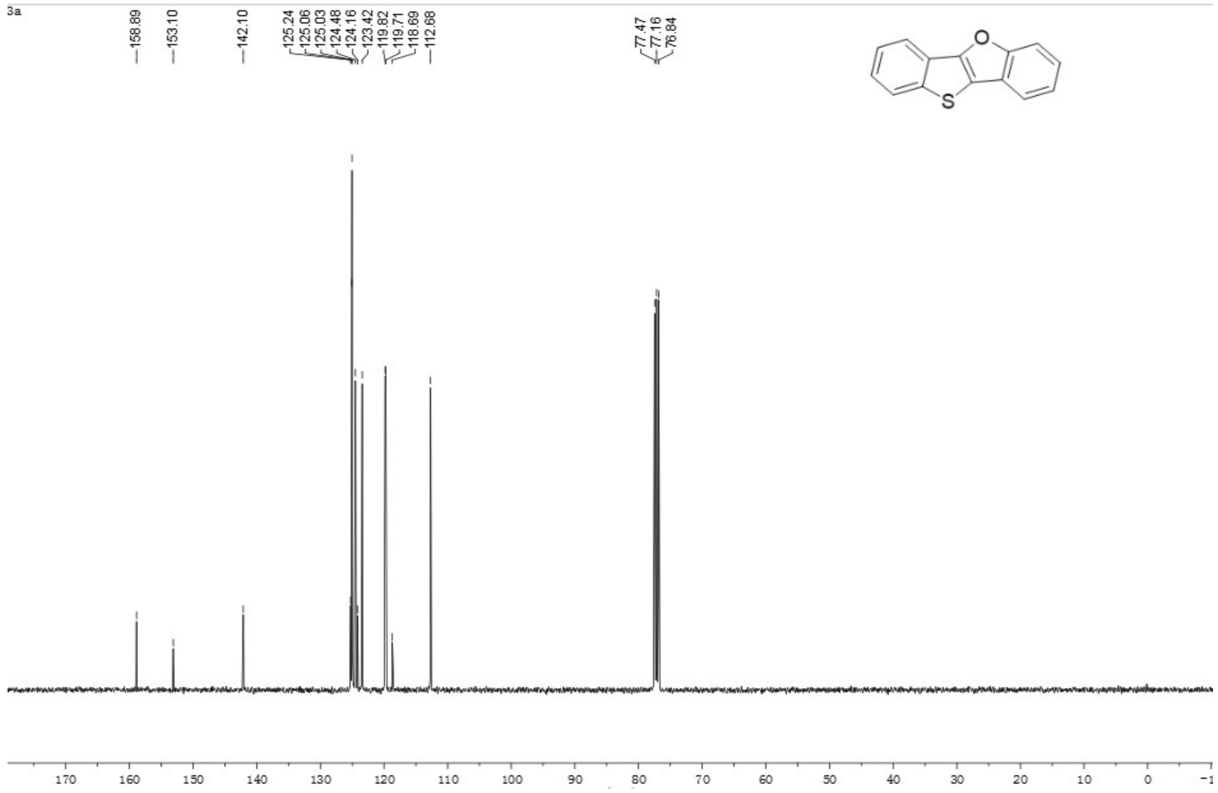

^{1}H NMR spectrum of **3b**

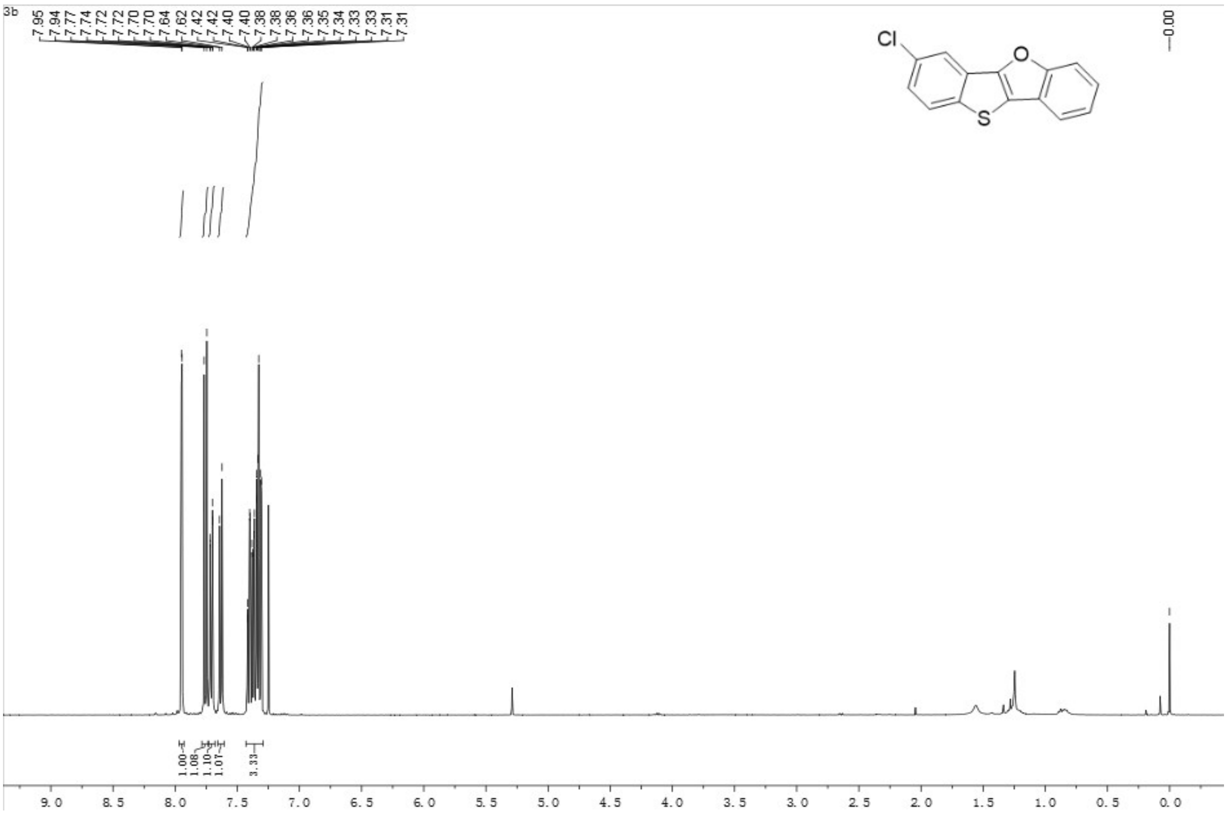

^{13}C NMR spectrum of **3b**

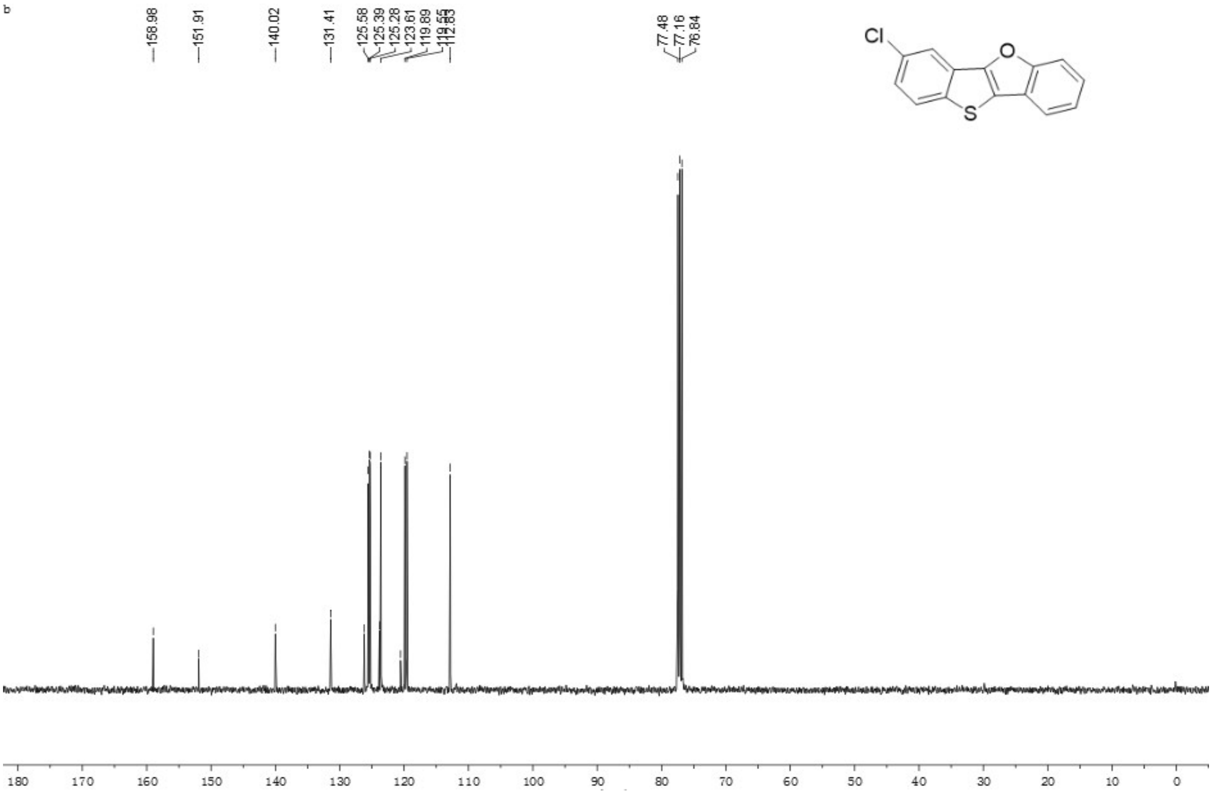

^{1}H NMR spectrum of **3c**

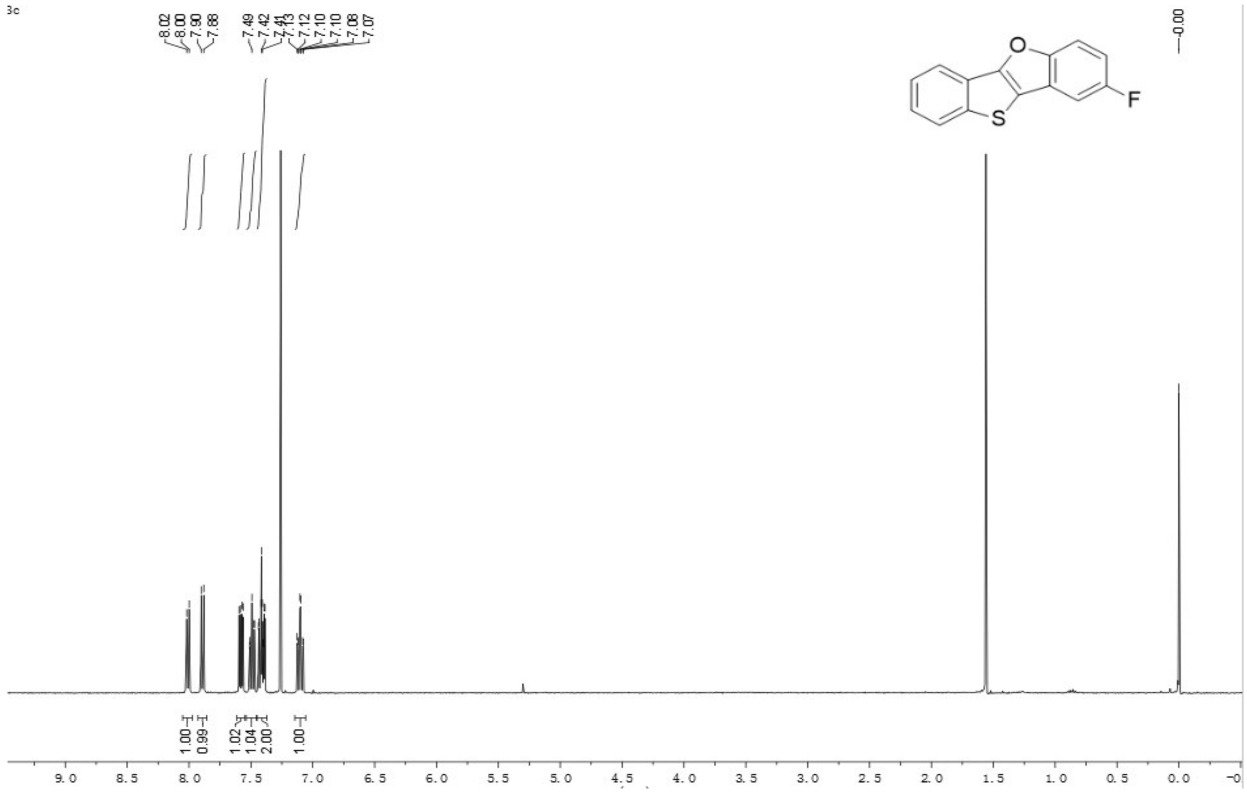

^{13}C NMR spectrum of **3c**

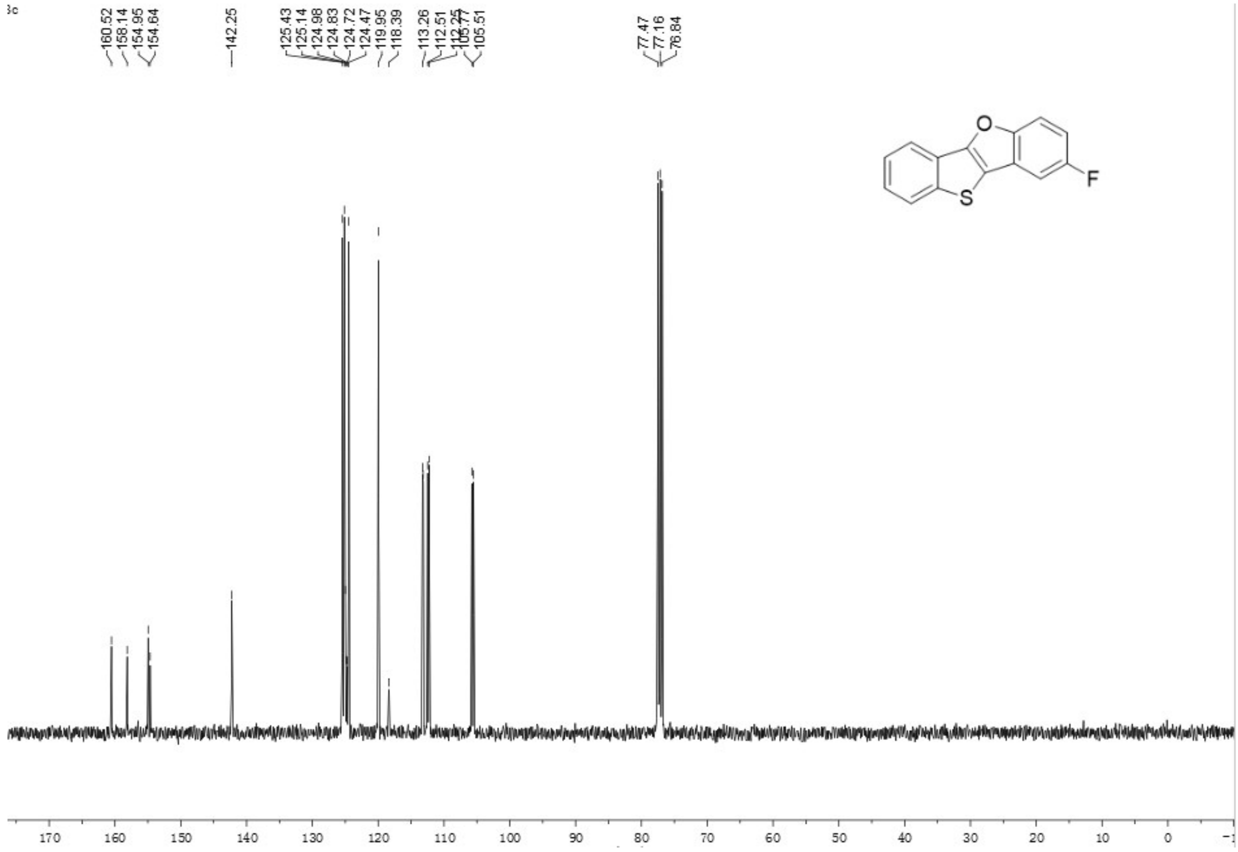

^{1}H NMR spectrum of **3d**

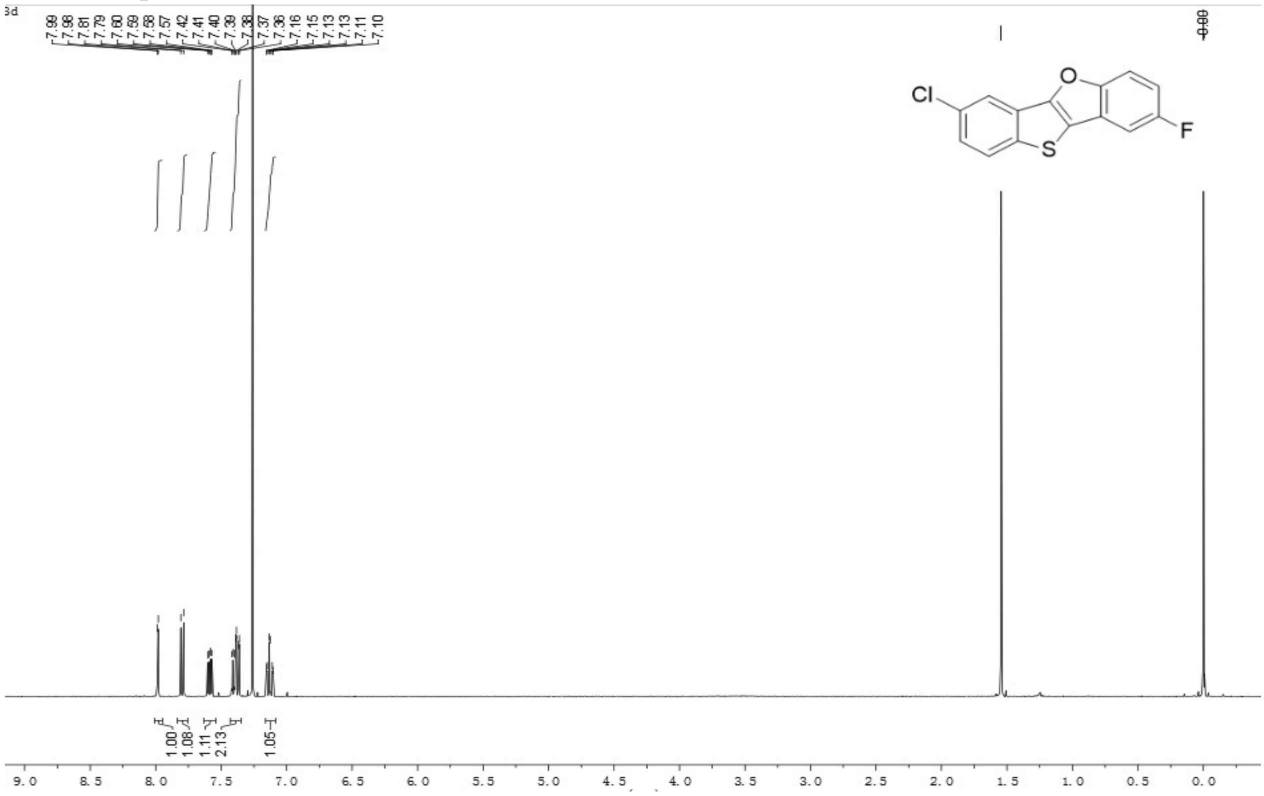

^{13}C NMR spectrum of **3d**

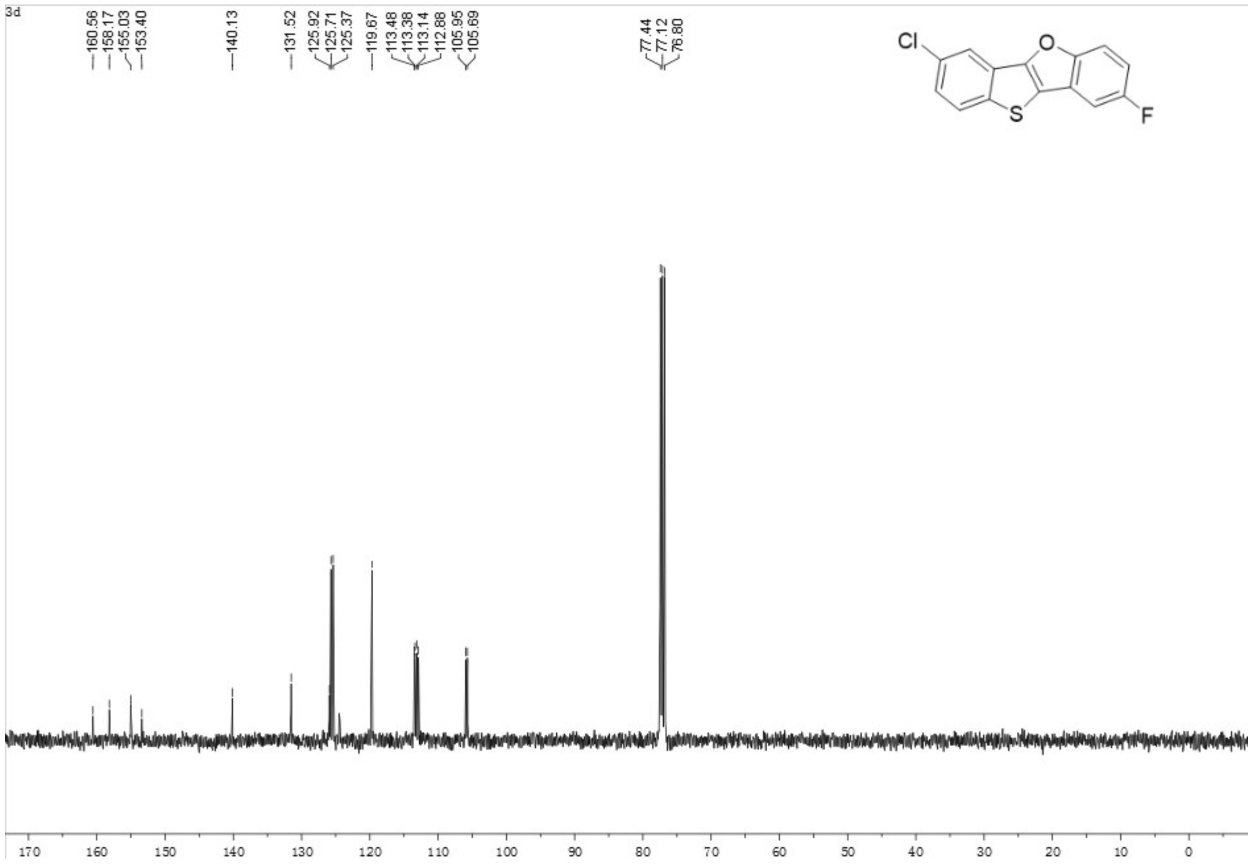

^{1}H NMR spectrum of **3e**

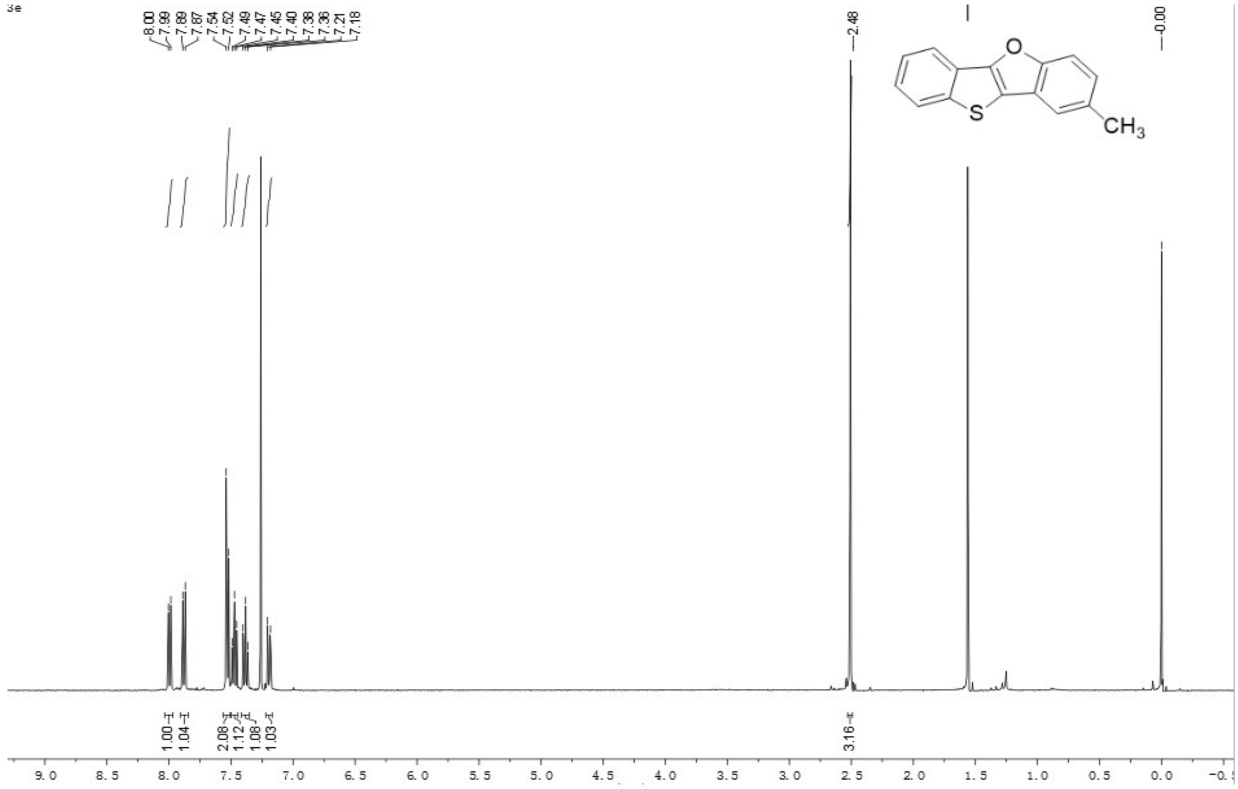

^{13}C NMR spectrum of **3e**

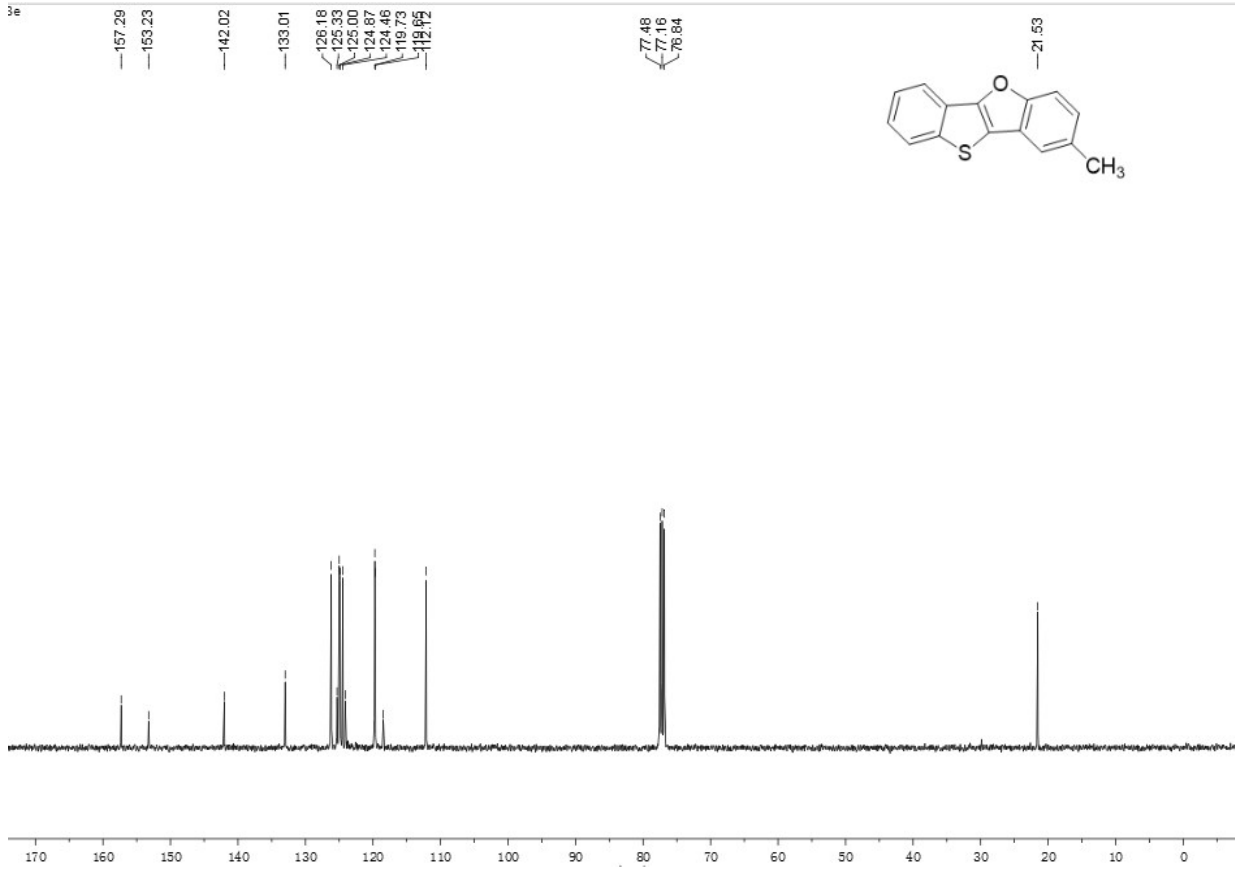

HRMS spectrum of **3e**

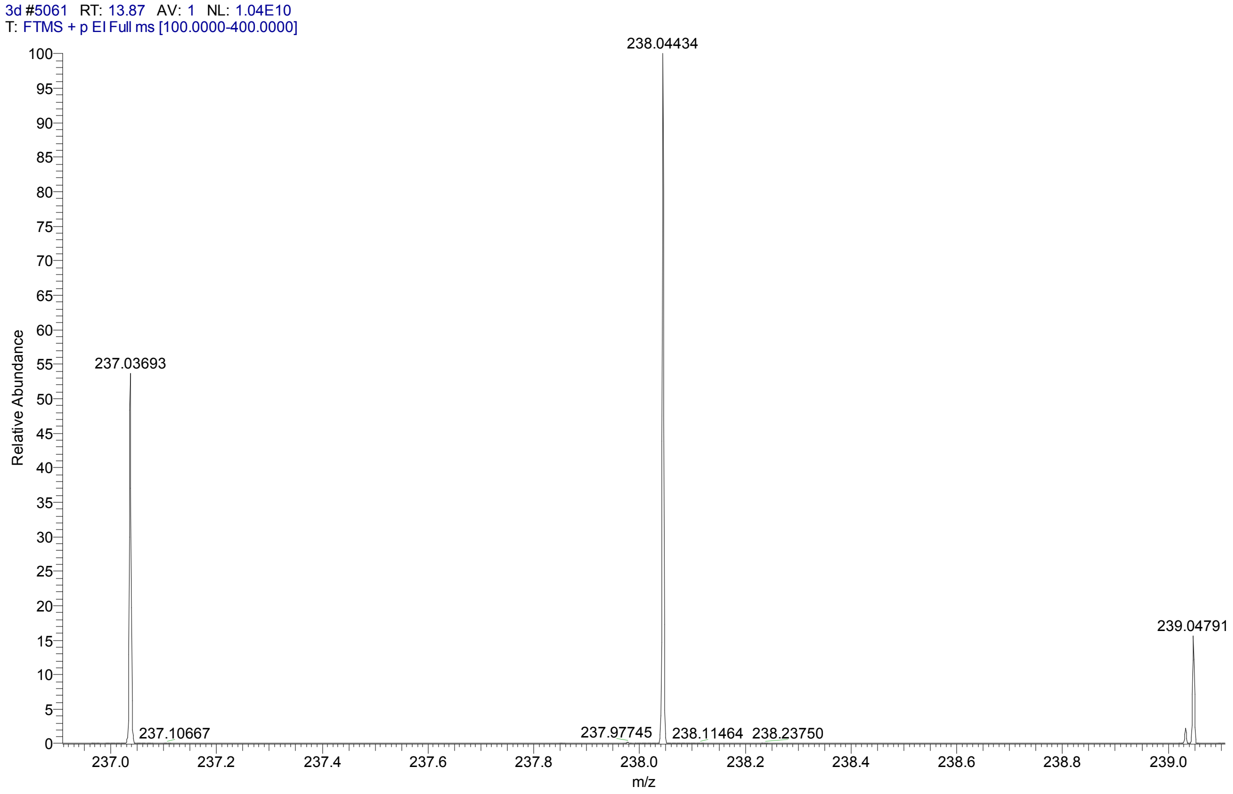

^{1}H NMR spectrum of **3f**

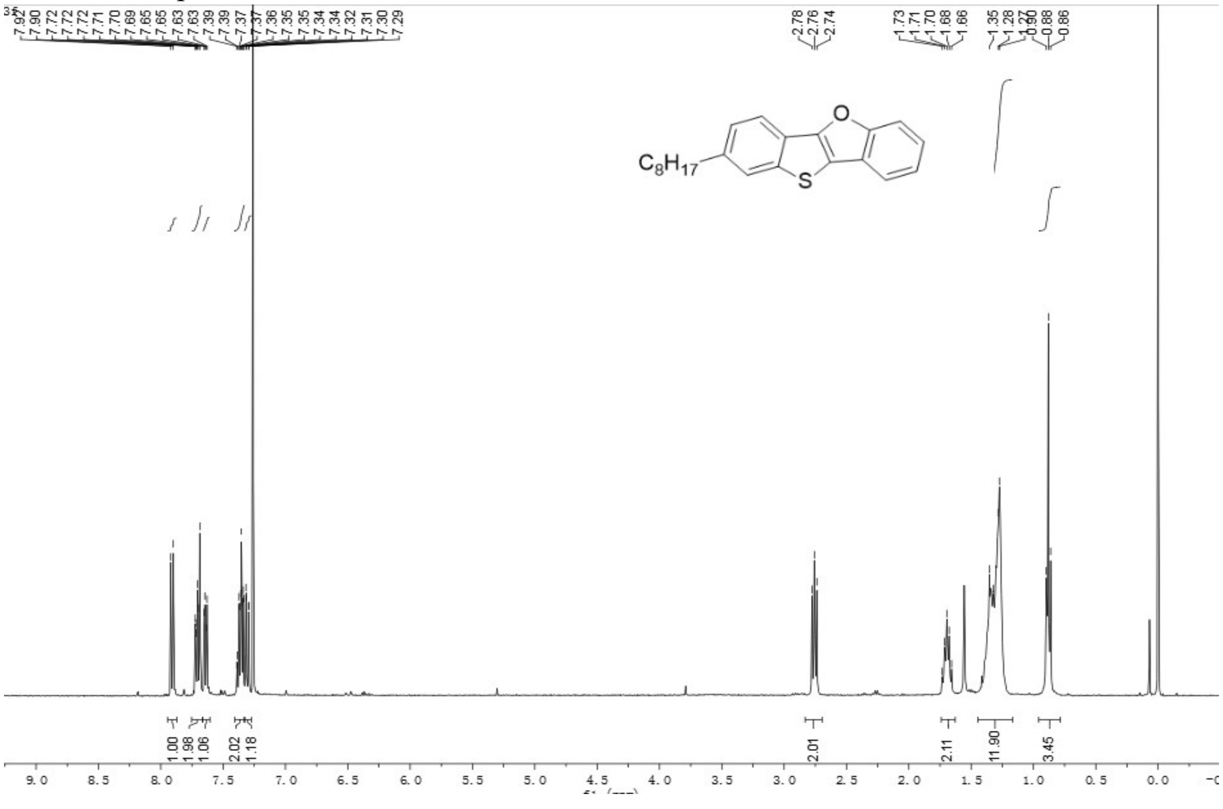

^{13}C NMR spectrum of **3f**

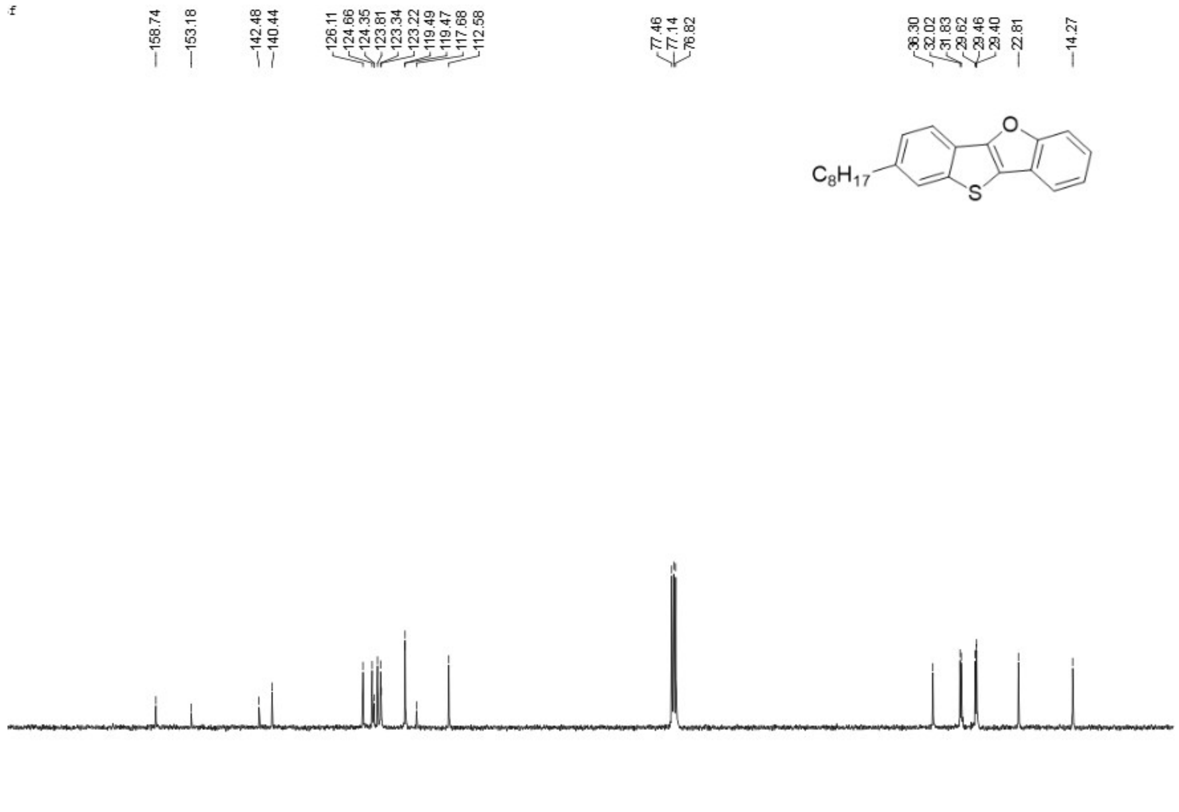

HRMS spectrum of **3f**

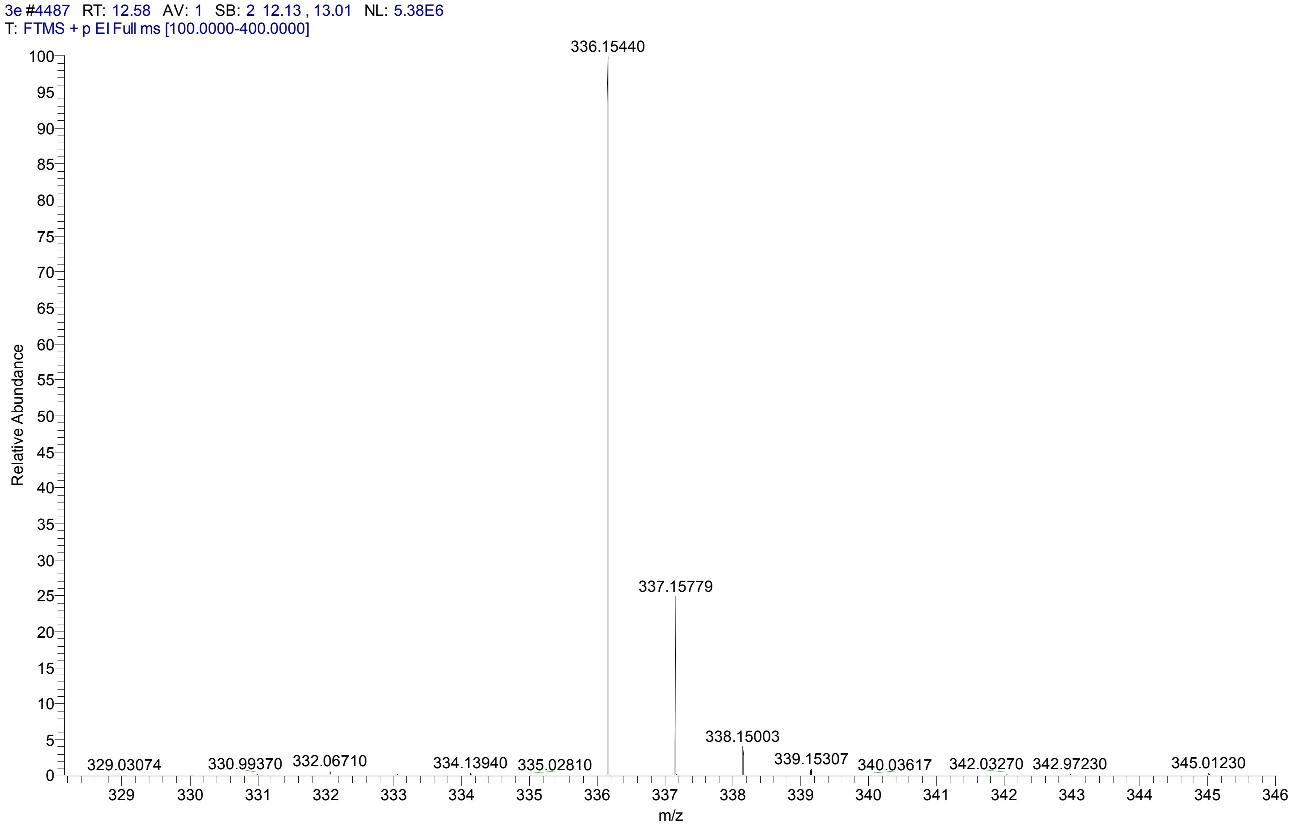

^{1}H NMR spectrum of **3g**

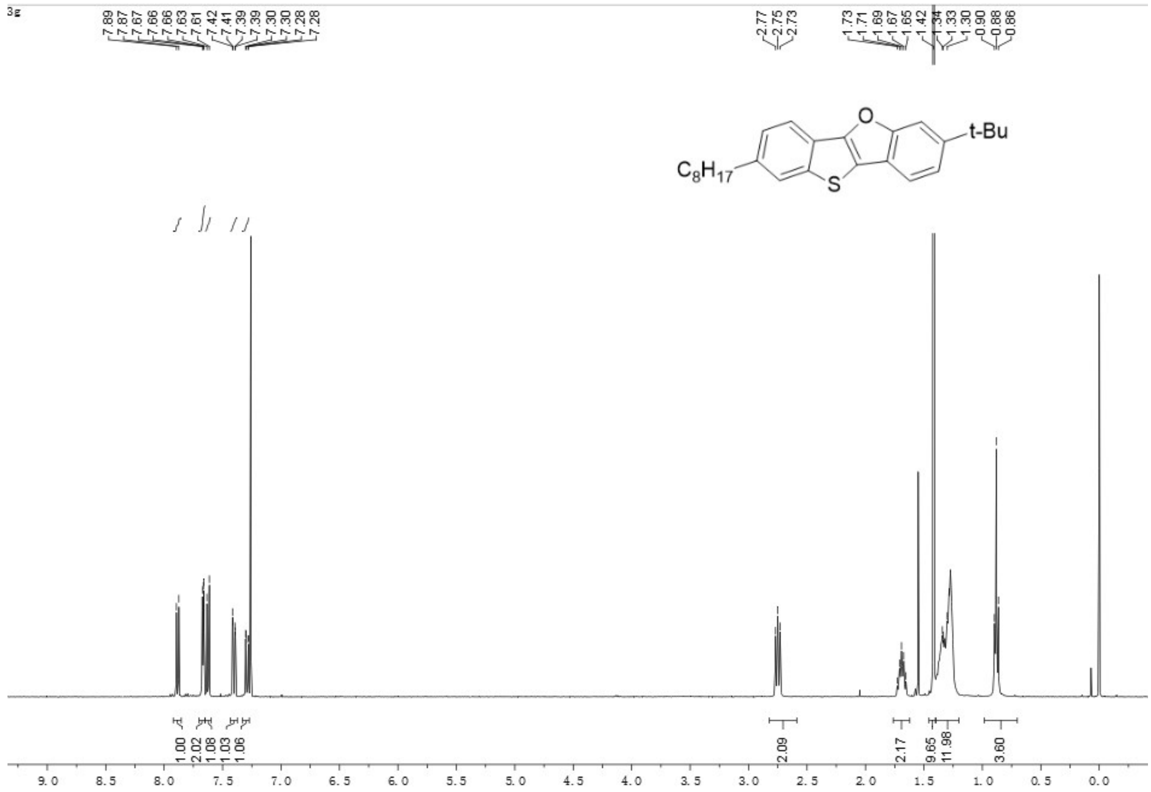

^{13}C NMR spectrum of **3g**

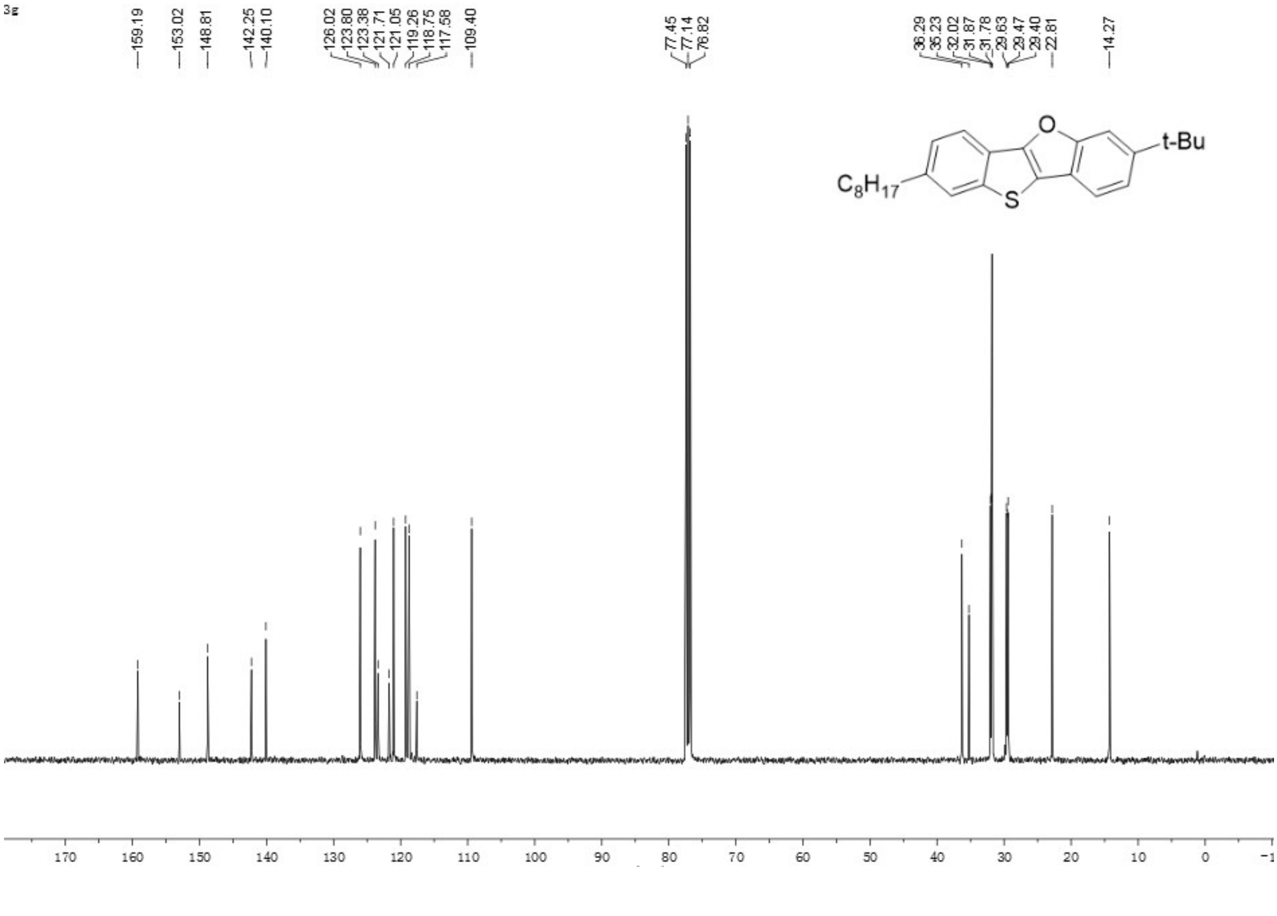

HRMS spectrum of **3g**

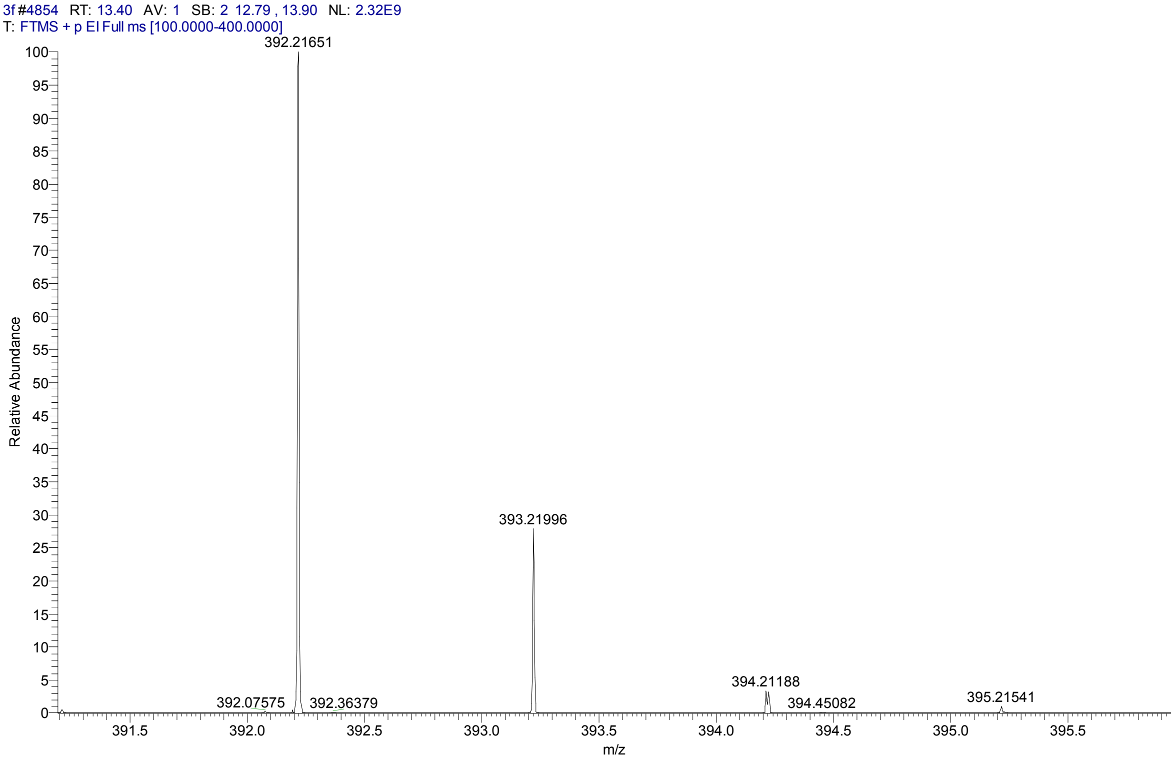

^{1}H NMR spectrum of **3h**

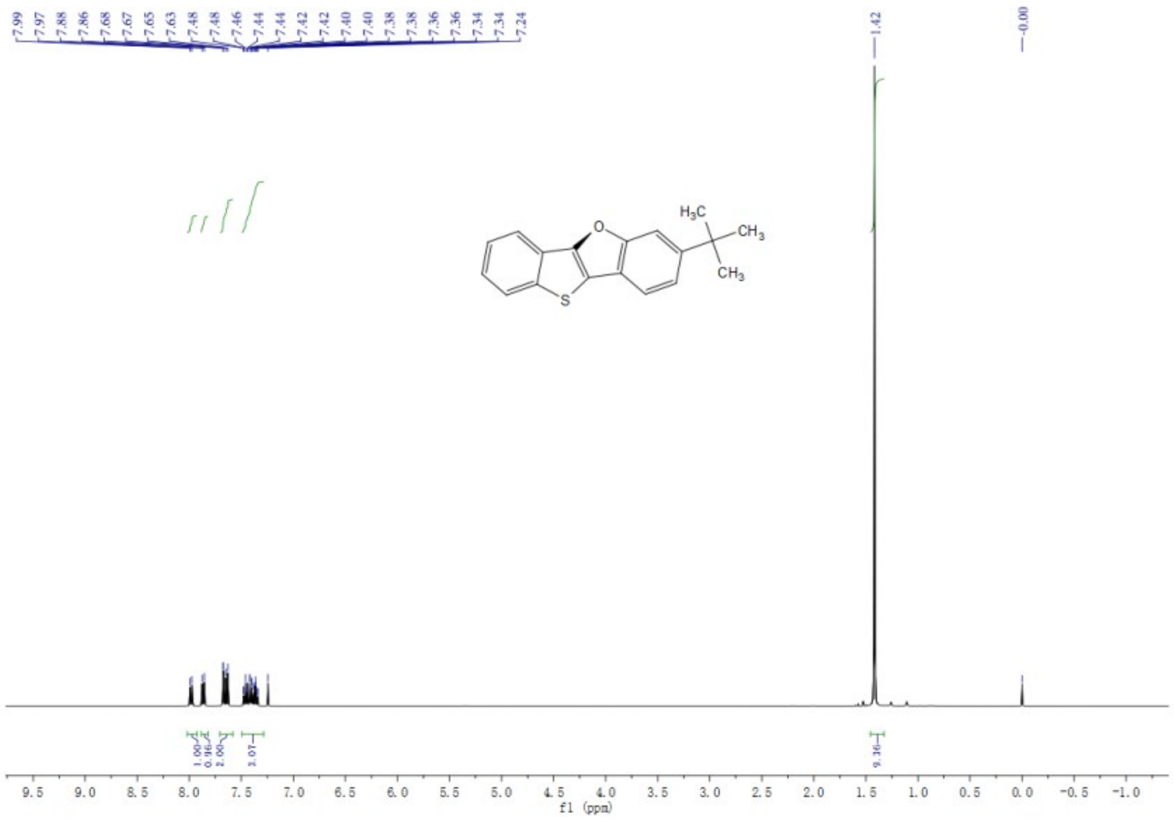

^{13}C NMR spectrum of **3h**

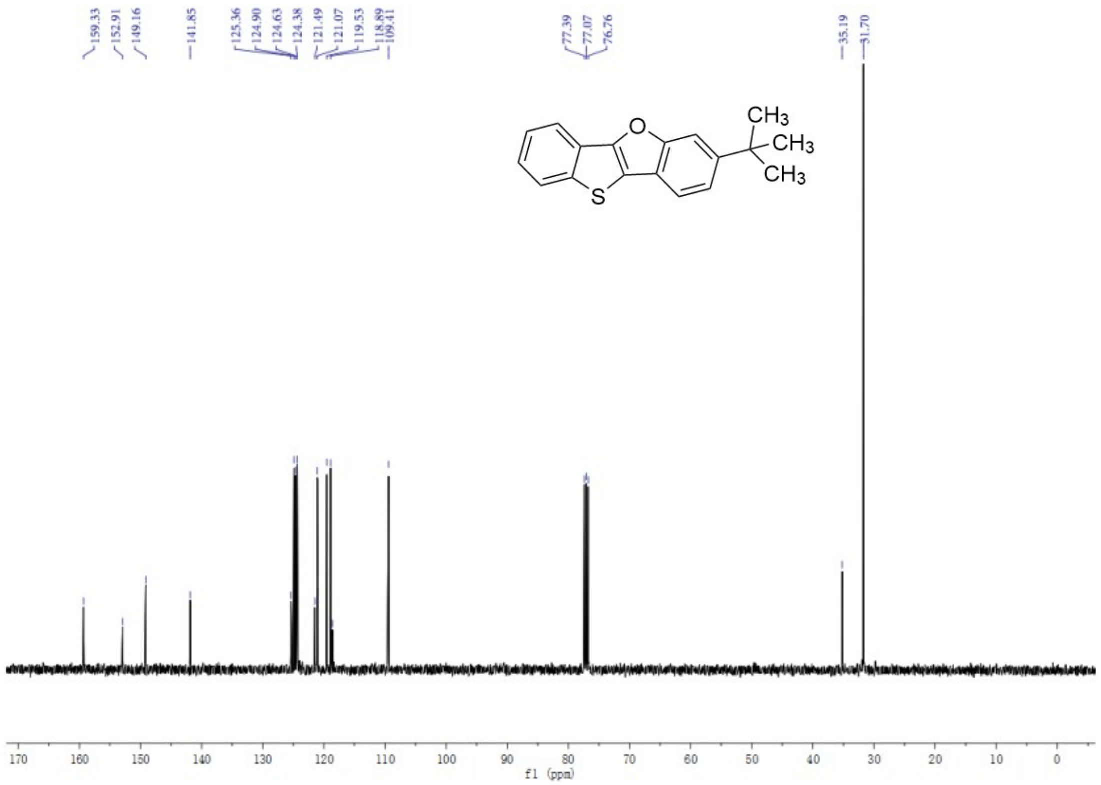

^{1}H NMR spectrum of **3i**

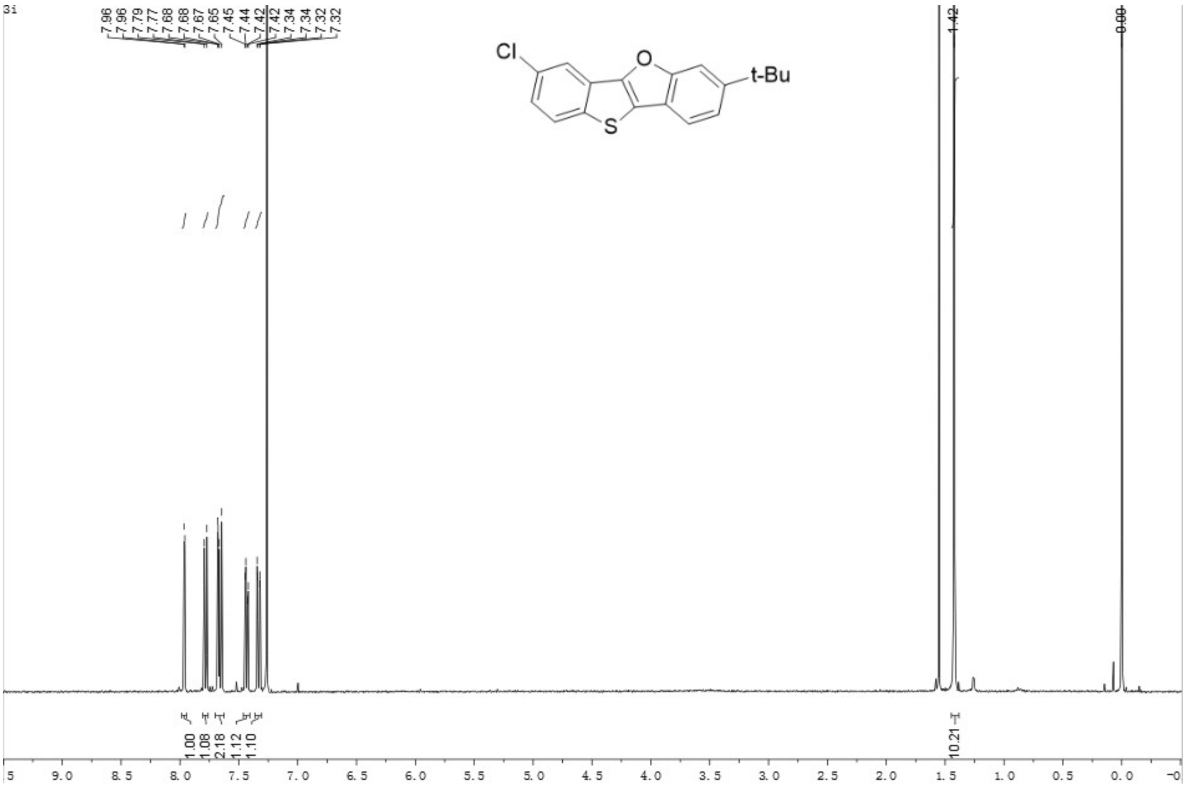

^{13}C NMR spectrum of **3i**

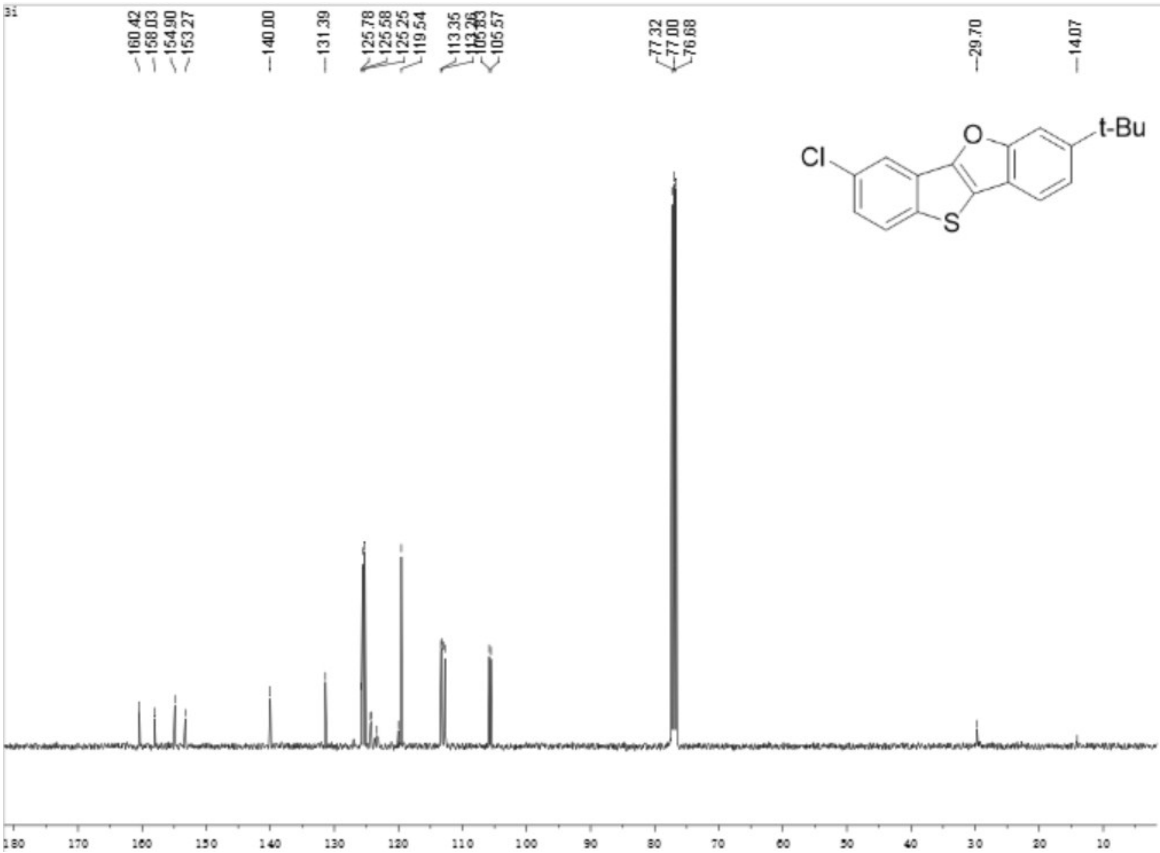

HRMS spectrum of **3i**

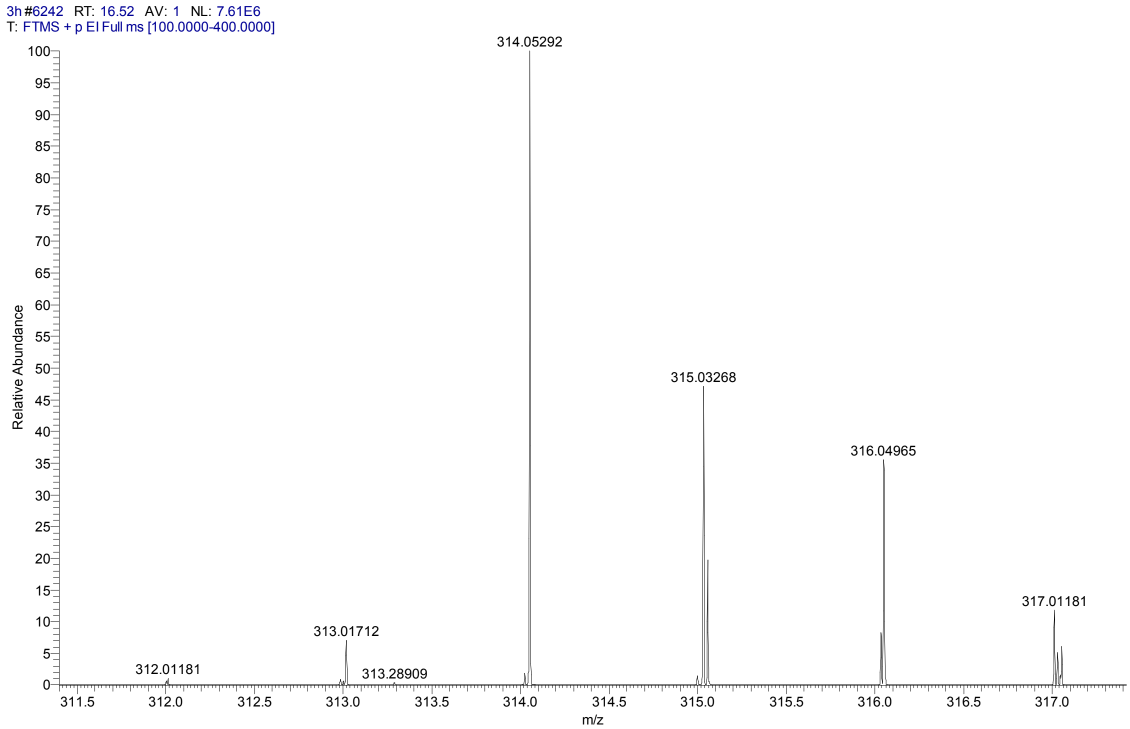

^{1}H NMR spectrum of **3j**

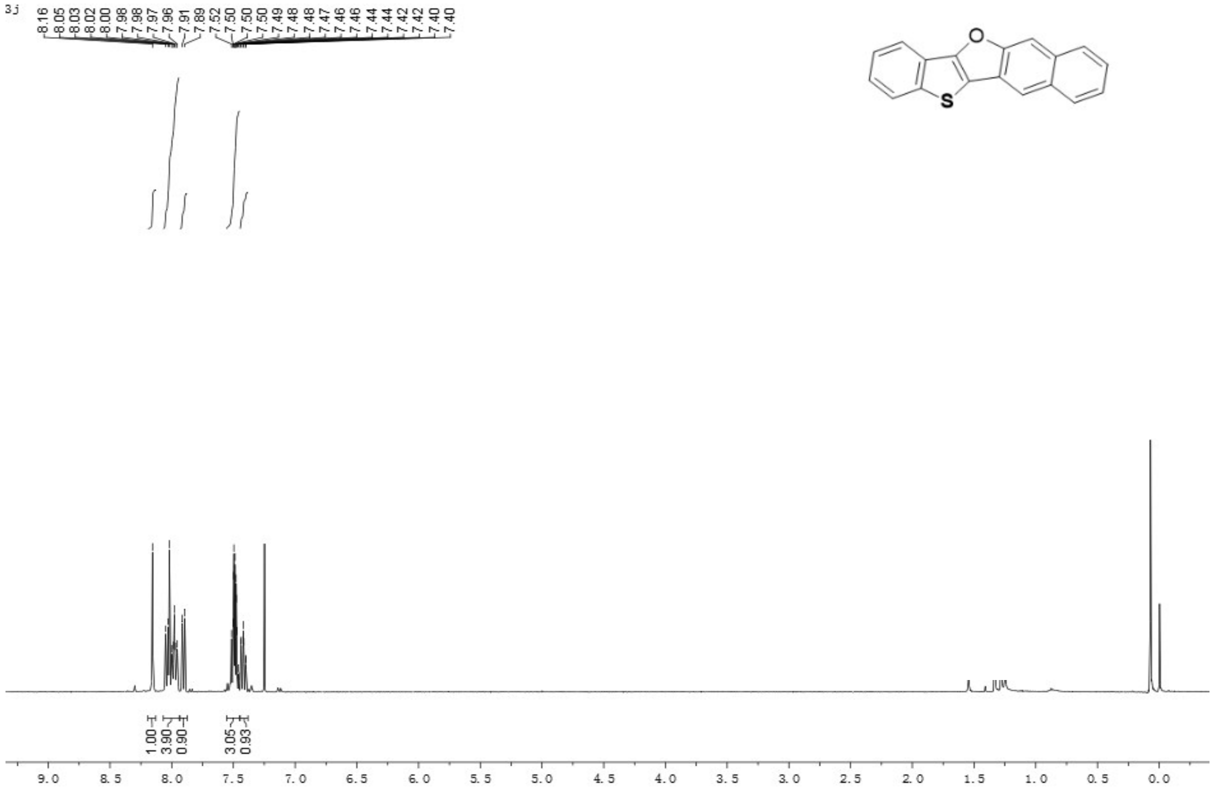

^{13}C NMR spectrum of **3j**

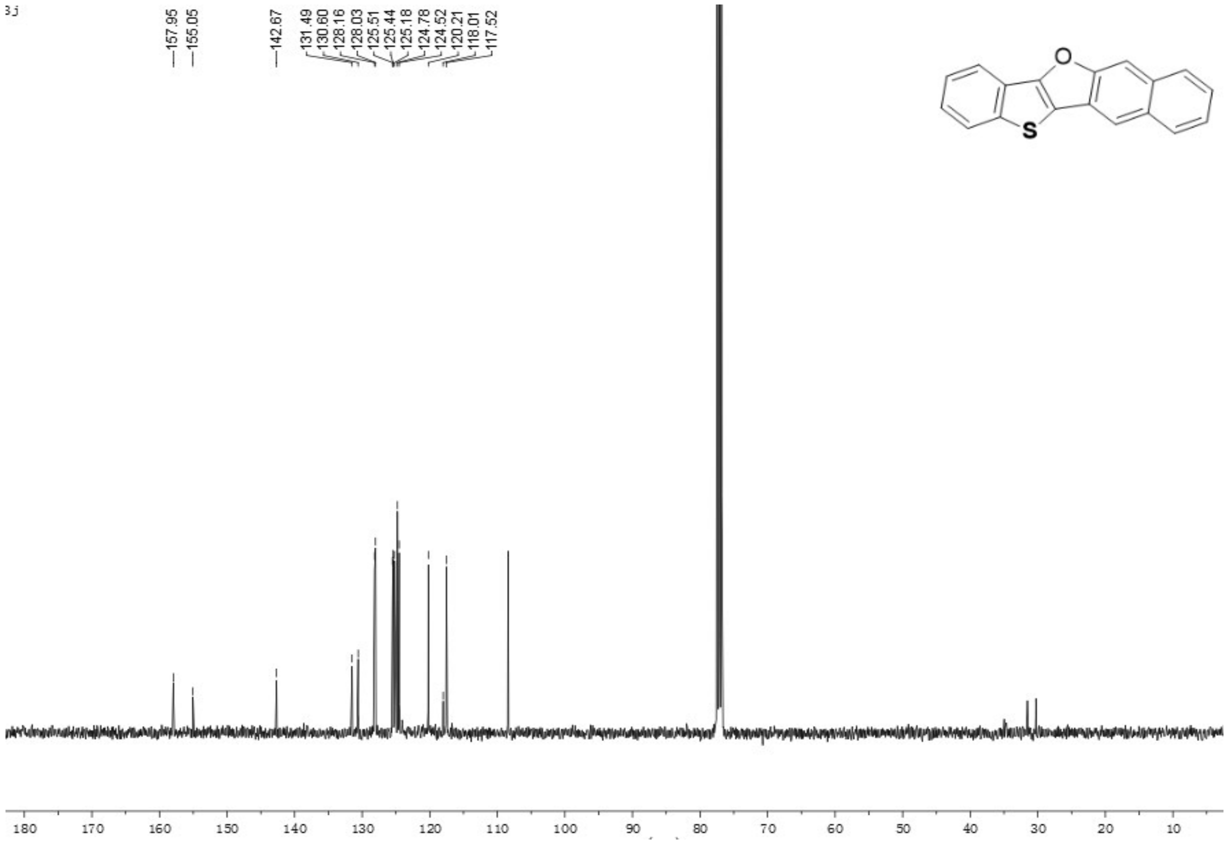

HRMS spectrum of **3j**

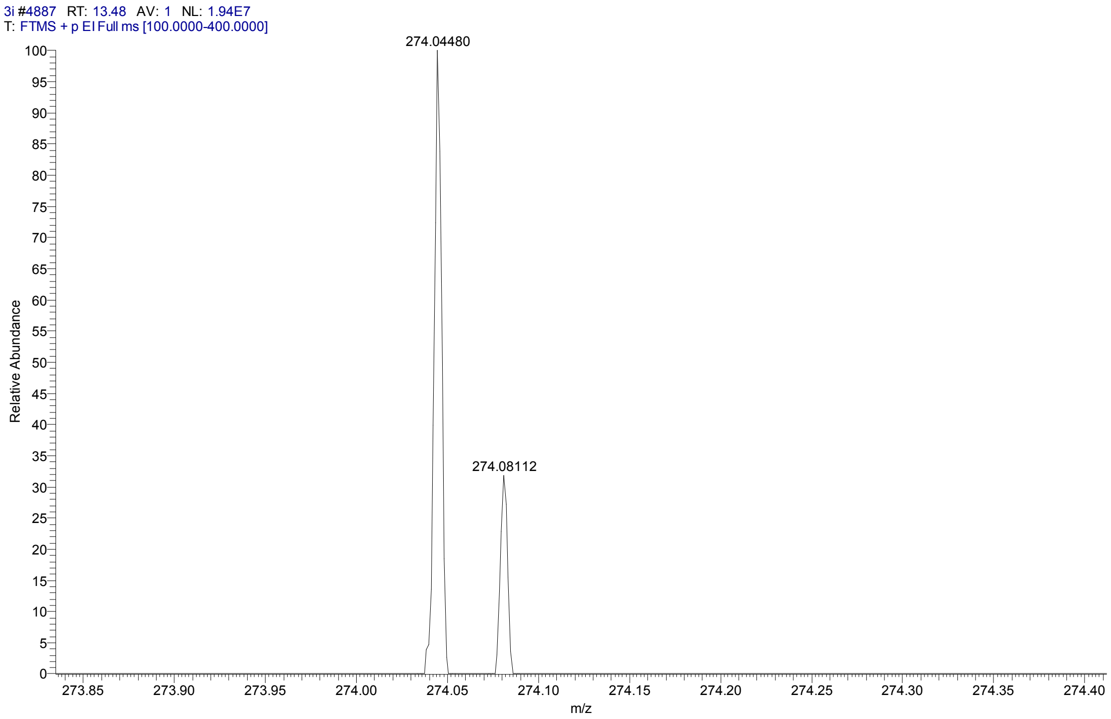

6. References

(1) Mo, F. Y.; Dong, G. B., *Science* **2014**, *345*, 68-72.

(2) (a) Lee, C.; Yang, W.; Parr, R. G., *Phy. Rev.* **1988**, *37*, 785-789. (b) Frisch, M.; Trucks, G.; Schlegel, H.; Scuseria, G.; Robb, M.; Cheeseman, J.; Scalmani, G., et al., *Gaussian 09, revision A.01; Gaussian, Inc.: Wallingfor, CT, 2009.*

(3) Chen, D. L. ; Yuan, D. F.; Zhang, C.; Wu, H.; Zhang, J.; Li, B. L.; Zhu, X. Z., *J. Org. Chem.* **2017,** *82*, 10920-10927.

(4) Zhang, H.; Ma, C.; Zheng, Z.; Sun, R.; Yu, X.; Zhao, J., *Chem. Commun. (Camb)* **2018,** *54*, 4935-4938.
